# The pesticide chlorpyrifos increases the risk of Parkinson’s disease

**DOI:** 10.1186/s13024-025-00915-z

**Published:** 2025-12-11

**Authors:** Kazi Md. Mahmudul Hasan, Lisa M. Barnhill, Kimberly C. Paul, Chao Peng, William Zeiger, Beate Ritz, Marisol Arellano, Michael Ajnassian, Shujing Zhang, Aye Theint Theint, Gazmend Elezi, Hilli Weinberger, Julian P. Whitelegge, Qing Bai, Sharon Li, Edward A. Burton, Jeff M. Bronstein

**Affiliations:** 1 Department of Neurology, David Geffen School of Medicine at UCLA, 710 Westwood Plaza, Los Angeles, CA 90095 USA; 2 Department of Epidemiology, University of California-Los Angeles, Los Angeles, CA 90095 USA; 3 Environmental and Molecular Toxicology Interdepartmental Program, University of California Los Angeles, Los Angeles, CA 90095 USA; 4 Pasarow Mass Spectrometry Laboratory, The Jane and Terry Semel Institute for Neuroscience and Human Behavior, David Geffen School of Medicine at UCLA, Los Angeles, CA 90095 USA; 5 Department of Neurology and Pittsburgh Institute for Neurodegenerative Disease, University of Pittsburgh School of Medicine, Pittsburgh, PA 15213- 3301 USA; 6 Geriatric Research, Education, and Clinical Center, Pittsburgh VA Healthcare System, Pittsburgh, PA 15240 USA

## Abstract

**Background:**

Pesticides as a class have been associated with an increased risk of Parkinson’s disease (PD), but it is unclear which specific pesticides contribute to this association and whether it is causal. Since chlorpyrifos (CPF) exposure has been implicated as a risk factor for PD, we investigated its association to incident PD and if this association is biologically plausible using human, rodent, and zebrafish (ZF) studies.

**Methods:**

The association of CPF with PD was performed using the UCLA PEG cohort (829 PD and 824 control subjects), the pesticide use report and geocoding the residence and work locations to estimate exposures. For the mammalian studies, 6 months old male mice were exposed to CPF by inhalation (consistent with human exposures) for 11 weeks and behavioral and stereological pathological analyses were performed. Transgenic ZF were utilized to determine the mechanism of CPF neurotoxicity.

**Results:**

Long-term residential exposure to CPF was associated with more than a 2.5-fold increased risk of developing PD. Mice exposed to aerosolized CPF developed motor impairment, dopaminergic neuron loss, microglial activation, and an increase in pathological α-synuclein (α-syn). Using ZF, we found that CPF-induced dopaminergic neuron loss was at least partially due to autophagy dysfunction and synuclein accumulation, as knocking down LC3 recapitulated the dopaminergic neuron loss and restoring autophagic flux or eliminating synuclein reduced neuronal vulnerability.

**Conclusions:**

CPF exposure is associated with an increased risk of developing PD and relevant exposures in animal models establish biological plausibility. In addition to establishing a new risk factor for PD, we identified new therapeutic targets for disease modification.

**Supplementary Information:**

The online version contains supplementary material available at 10.1186/s13024-025-00915-z.

## Introduction

Parkinson’s disease (PD) is a slowly progressive neurodegenerative disorder manifested by motor dysfunction and cognitive decline. The primary pathological hallmarks of PD are the selective loss of dopaminergic (DA) neurons in the substantia nigra pars compacta (SNpc), the development of fibrillar cytoplasmic inclusions, also known as Lewy bodies and Lewy neuritis, and inflammation. The major component of Lewy bodies and neurites is misfolded, highly ubiquitinated and phosphorylated α-synuclein (α-syn). The etiology of PD appears complex and involves the interaction of both genetic and environmental factors. A minority of PD cases are caused by mutations in one of several genes, including *SNCA*, *LRRK2*, *GBA*, *VPS35*, *RAB32*, and *PINK1*, that code for proteins involved in proteostasis, mitochondrial function, and inflammation [1–3]. The etiology of the majority of PD cases is not known but likely involves environmental factors. Pesticide exposure as a class of toxicants is one of the strongest and best documented risk factors associated with an increased risk of PD, but few individual chemicals have been identified that confer increased risk [4, 5]. It is essential to identify specific pesticide in order to determine whether the associations are causative and what is the underlying mechanism of toxicity.

Several studies have investigated the role of environmental toxicants in the development of PD [4, 6], but there are challenges in determining whether an association is causal. PD develops over decades, and exposure assessment should cover the time before pathology starts. Once a toxicant has been associated with altered risk, further studies are necessary to determine if the disease pathology can be recapitulated by relevant exposures in animal models and the mechanisms by which they act (i.e. biological plausibility). For example, rotenone is one of the very few toxicants that is associated with an increased risk of developing PD and exposure to rotenone results in motor abnormalities, α-syn inclusions, DA neuronal loss, and inflammation [5, 7].

Chlorpyrifos (CPF; O,O-diethyl O-3,5,6-trichloro-2-pyridyl phosphorothioate) is a broad-spectrum organophosphate pesticide extensively used in agriculture to control insect pests. This pesticide acts by inhibiting acetylcholinesterase, leading to the death of insects through its neurotoxicity [8]. CPF was widely used in the US until 2000 when the EPA banned its use indoors due to its neurodevelopmental risks to children [9]. It continues to be used in agriculture and is generally applied by spraying. Thus, inhalation of CPF is the primary means of human exposure. Although the neurodevelopmental risks of CPF have been well documented, few studies have investigated its association with the risk of developing PD and the mechanism by which it acts [10–14].

Here, we report that exposure to CPF is associated with an increased risk of developing PD in a large community-based case-control study. Mice exposed to CPF using a novel inhalation method that recapitulates human exposures, caused impaired motor behavior, loss of DA neurons, increased pathological α-syn, and inflammation. Using transgenic zebrafish (ZF), we found that CPF was toxic to neurons by disrupting autophagic flux and was dependent on γ1-synuclein (γ1-syn), the closest functional homologue to human α-syn. Together, these studies strongly implicate exposure to CPF as a risk factor for developing PD and modulators of autophagy is a promising therapeutic target.

## Methods

### PEG study population

To assess CPF and PD associations, we used the Parkinson’s Environment and Genes (PEG) study (*n* = 829 PD patients; *n* = 824 controls). The PEG study is a population-based case-control studies conducted in three agricultural counties in central California (Kern, Fresno, and Tulare) [14, 15]. Participants were recruited in two waves: Wave 1 (PEG1): 2000–07, *n* = 357 patients, *n* = 400 population-based controls; and Wave 2 (PEG2): 2009–15, *n* = 472 patients, *n* = 424 population-based controls. Patients were enrolled early in the disease course [mean PD duration at baseline, 3.0 years (SD = 2.6)] and all were seen by University of California, Los Angeles (UCLA) movement disorder specialists for in-person neurological examinations, many on multiple occasions, and confirmed as having probable idiopathic PD [16, 17]. As shown in Table 1, PD patients were on average slightly older than controls and a higher proportion were men, had European ancestry, and were never smokers.

**Table 1:** Study population characteristics

Variable: Mean (SD) or* n* (%)	PD patients (*n*=829)	Controls (*n*=824)
Age	67.7 (10.6)	65.9 (11.6)
Male Sex	524 (63.2)	383 (46.5)
Years of Education	14 (4.6)	14 (4.0)
European Ancestry	634 (76.5)	569 (69.2)
Non-European Ancestry	195 (23.5)	253 (30.8)
White	631 (76.5)	569 (69.0)
Latino	137 (16.6)	155 (18.8)
Asian	22 (2.7)	26 (3.2)
Other	39 (4.7)	74 (9.0)
Never Smoker	449 (54.4)	397 (48.2)
Former Smoker	345 (41.8)	331 (40.2)
Current Smoker	31 (3.8)	96 (11.7)

### CPF exposure assessment

**Fig. 1:**
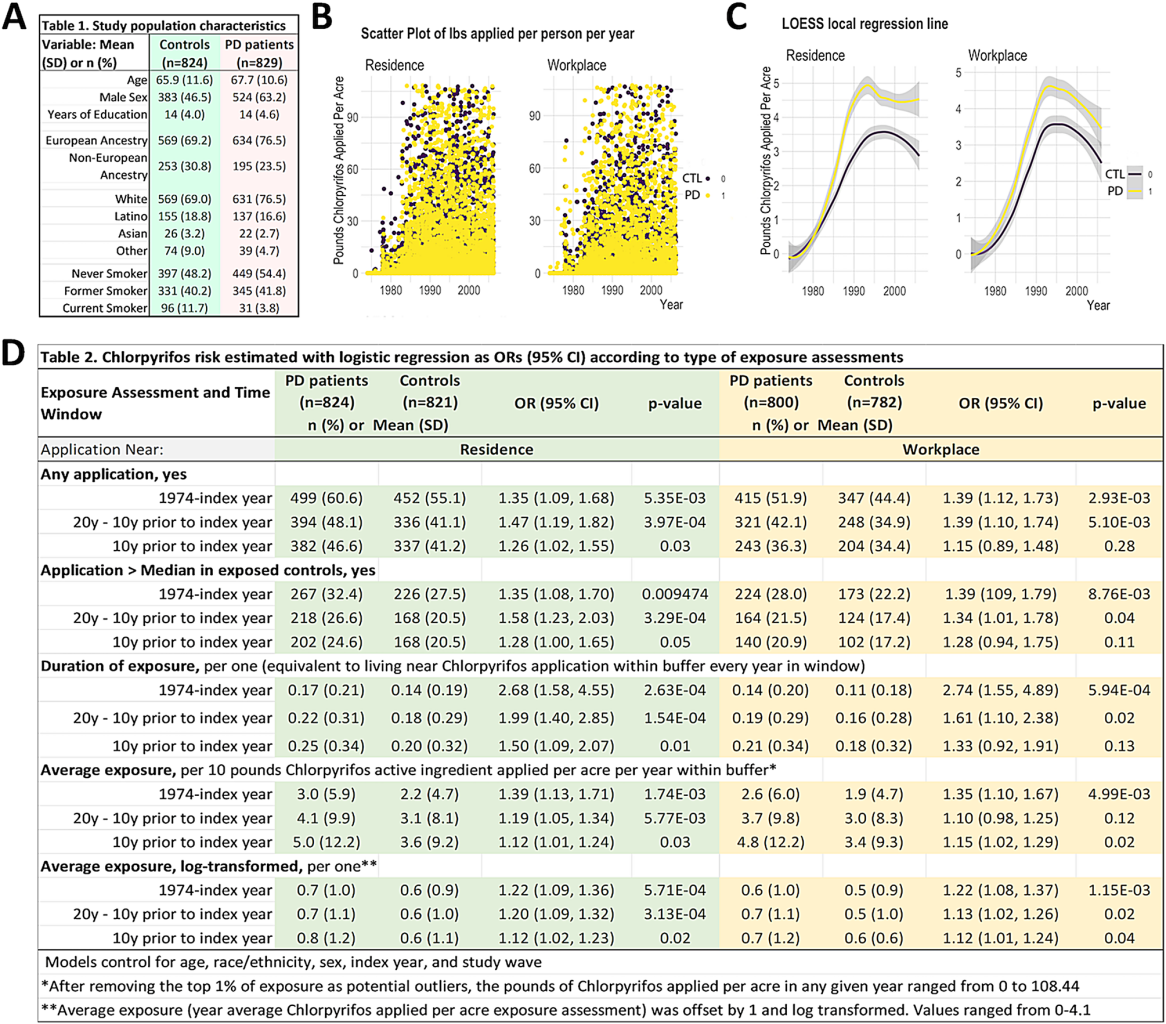
CPF exposure is associated with an increased risk of PD. (**A**) Demographic characteristics of PD patients and non-PD participants (**B**) Scatter plot of pounds active ingredient applied per acre for each participant each year within 500 m of residential address and workplace address. (**C**) Smoothed trend line from loess local regression based on data shown in plots. Lb, pounds; LOESS, locally estimated scatterplot smoothing. (**D**) CPF risk estimated with logistic regression as ORs (95% CI) according to the type of exposure assessments

We estimated ambient exposure due to living or working near agricultural CPF application, using pesticide use report (PUR) pesticide application data within a geographical information system (GIS)-based model [18]. Since 1972, California law mandates the recording of commercial pesticide use in a database maintained by the California Department of Pesticide Regulation (CA-DPR) that includes all commercial agricultural pesticide use by pest control operators and all restricted pesticide use until 1989, and afterwards (1990–current), all commercial agricultural pesticide use. This database records the location of applications, which was linked to the Public Land Survey System (PLSS), and the poundage, type of crop, and acreage a pesticide has been applied on, as well as the method and date of application. We combined the PUR with maps of land use and crop cover, providing a digital representation of historical land use, to determine pesticide applications at specific agricultural sites [19]. PEG participants provided lifetime residential and workplace address information, which we geocoded in a multi-step process [20]. As shown in Fig. 1, the scatter plot of the data is accompanied by smoothed trend lines based on local regressions (Fig. 1B-C). We determined the pounds of CPF applied per acre within a 500-meter buffer of the latitude and longitude representing each residential and workplace address per year since 1974, weighting the total poundage by the proportion of acreage treated (lbs/acre). For our study participants, this resulted in 12,904 annual records for residential and 8,968 for occupational site CPF exposure. After we identified and removed several extreme outliers (values >99th percentile of the distribution), the resulting data ranged from 0 to 108.44 lbs/acre. We also estimated exposure to paraquat, diazinon, and glyphosate in a similar manner for co-exposure adjustment.

### Mouse strain

Six-month-old, 24 male wild-type mice on C57BL/6 background weighing 29–33 g were purchased from the Jackson Laboratory, Sacramento, California, USA. The mice were housed in a vivarium with 12/12 hours of light and dark cycles. UCLA Animal Research Committee approved all experiments using animals in accordance with the US National Institutes of Health guidelines.

### CPF exposure by inhalation

Sixteen 26-week-old male mice were exposed to aerosolized CPF (Sigma #45395) and another 16 were exposed to room air passed through the control vehicle containing 2% ethanol in identical chambers using custom-built whole-body chamber systems (CH Technologies, Westwood, NJ). The chamber holds up to 20 mice and is connected to a Blaustein Jet Atomizer, which produces aerosols that are tightly controlled with a Lab Flow C Control box. Aerosols were generated from solutions containing CPF (0.2 mg/mL) in 75% ETOH at a flow rate into the atomizer of 16 mL/hr while aerosol generator air flow was 1.5 L/min and dilution air flow was 4.5 L/min. The concentration of CPF initially in the chamber was 76.63 µg/m^3^ and was gradually increased to 300 µg/m^3^. Mice were exposed 5 days/wk. for 6 h./day for 11 weeks with total exposures of 0.65–2.9 mg/m^3^/day.

### Behavioral testing

Behavioral testing was performed prior to CPF exposures and 3 days after the final day of exposure to wash out any residual effects of the pesticide and vehicle.

*Rotarod Test*: Rotarod testing was carried out as previously described [20, 21]. Mice were trained to run on the rotarod (Bioseb, Park, FL USA) with four 5-minute trials at 12 rotations per minute (rpm) separated by five minutes. One hour later, mice were placed on the rotarod at 4 (rpm) and the speed was linearly accelerated to 40 rpm over 300 s. The latency to fall and speed at which the mice fell were recorded. The mean latency to fall was calculated from two consecutive trials in each mouse.

*Wire Hang Test*: Wire hang testing was carried out according to a modified protocol as previously described [20, 22]. Briefly, the mice were placed on the top of a wire mesh (wire diameter 0.047” with 0.45” openings) connected to a stepper motor controlled by an Arduino microcontroller, with speed set to 15 rpm. At the start of the trial, the wire mesh was sequentially rotated 36 degrees in one direction then the other, causing the mice to grip the wires, and then flipped 180 degrees so mice were suspended upside down. Latency to fall was measured using an infrared sensor placed below the wire mesh. If mice did not fall within 15 min, the trial was terminated.

*Open Field Test*: Open field testing was performed using a custom-made acrylic square arena (16” x 16” x 12”) with a grey floor that was evenly illuminated to 100–200 lux [20, 23]. During the trial, the mice were allowed to explore freely for 10 minutes while being videotaped. A deep neural network was trained on behavior videos to identify arena boundaries and anatomical markers on mice (nose, ears, and tailbase) using DeepLabCut [20, 24]. The trained model was then used to automatically extract marker positions during each video frame. Mouse position was calculated as the centroid of the polygon between the mouse ear and tailbase markers, and custom-written code in MATLAB was used to calculate mean velocity, distance traveled, and time in the center of the arena (defined as a square 20% of the arena diameter away from each wall).

### Liquid chromatography-tandem mass spectrometry LC-MS/MS

Commercially available CPF was acquired from Chem Service Inc. Diethyl-d10 (CPF-d10) is obtained from CDN Isotopes. Methanol and acetonitrile optima LC/MS grade were purchased from Thermo Fisher Scientific. Standards, controls, and brain tissue samples were prepared in 2 ml bead-beating tubes in duplicates. A five-concentration point (100, 200, 400, 800, 1600 ng/50 mg) standard curve and two control samples at low (200 ng/50 mg) and high (1600 ng/50 mg) concentrations were utilized for compound quantification. Standards, controls, and brain tissue samples stored at -80 °C were left to thaw at ambient temperature. To each tube were added six 1.4 mm and three 2.8 mm ceramic beads, 400 ng diethyl-d10 stock solution as an internal standard (IS) prepared in acetonitrile, and 500 mL of a 4:1 mixture of acetonitrile/ deionized water for extraction. The samples were homogenized in the bead-beater for 30 s and centrifuged at 16,000 x g for five minutes. The supernatant was transferred to new, respectively labeled 1.5 ml microcentrifuge tubes, and samples were dried down in a low-speed vacuum concentrator. The samples were then reconstituted in 100 µl of a methanol/water (v/v 70/30) mixture, vortexed thoroughly, and centrifuged at 16,000 x g for five minutes. The supernatant was transferred to HPLC vials, from which 15 µl was injected into the mass spectrometer system for analysis. Analysis of the analyte concentration was done at the UCLA Pasarow Mass Spectrometry Lab. A targeted LC-MS/MS multiple reaction monitoring (MRM) acquisition method was developed and optimized on a 6460 Agilent Technologies triple quadrupole mass spectrometer. The mass spectrometer was coupled to a 1290 Infinity HPLC system (Agilent Technologies) through a Scherzo SM C-18 analytical column (3 μm 50 × 2 mm UP). For compound elution, a mixture of solvent A (water/formic acid v/v 100/0.1) and solvent B (acetonitrile/formic acid v/v 100/0.1) was used as a mobile phase combined with a linear gradient (min/%B: 0/0, 2/5, 6/100, 7/100, 8/5, 10/5). For the MRM method, the transition from the precursor ion m/z 349.9 to the production m/z 97 isolated for CPF was monitored in positive ion mode at a specific LC retention time. The standard curve was made by plotting the known amount of analyte per standard vs. the ratio of measured chromatographic peak areas corresponding to the analyte over that of the IS (analyte/IS). The trendline equation was used to calculate the absolute concentrations of the analyte in brain tissue.

### Mouse immunohistochemistry

After perfusion and fixation of six CPF-exposed and six control mice, brains were embedded in paraffin blocks, cut into 6 mm sections and mounted on glass slides as previously described with some modifications [20, 25]. Briefly, samples containing slides were deparaffinized and rehydrated, followed by immersion in 88% formic acid for 5 min to enhance antigen detection. Then the sections were treated with 5% hydrogen peroxide (Sigma#H1009) in methanol (Fisher Scientific#A454-4) for 30 min to quench endogenous peroxidases. The sections were then blocked in 0.1 M Tris with 2% fetal bovine serum (Tris/FBS) for 5 min. Both TH primary and secondary antibodies were diluted in Tris/FBS. Samples were then incubated with primary antibody (Abcam#AB76442) overnight at 4 °C. Following washing, sections were incubated for 1 h with species-specific biotinylated secondary antibody (VectorLabs#BA-9010) and then for 1 h with VECTASTAIN^®^ Elite^®^ ABC-HRP Kits (VectorLabs#PK-6100). After rinsing with 0.1 M Tris, the slides were incubated for 4 min with DAB reagent (VectorLabs#SK-4105). In subsequent steps, slides were rinsed and washed, counterstained in Hematoxylin (Fisher Scientific#6765001), then dehydrated and cover slipped. Slides were scanned into digital format on a Lamina scanner (Perkin Elmer) at 20x magnification. Digitized slides were then used for quantitative pathology. All TH positive neurons in the midbrain (SNpc and VTA) were counted in a blinded manner.

In a separate group of 6 pairs of mice, brains were quickly removed after perfusion with PBS and euthanasia and cut in half sagittally. Half of the brains were quickly frozen for biochemical studies, and the other half were fixed in 4% paraformaldehyde for at least 24 h. Following dehydration in 30% sucrose, they were embedded in OCT (Sakura Finetek#4583) and 40 µM sections were collected. The sections were blocked with 3% FBS and 3% bovine serum albumin (Sigma#B-4287) and incubated overnight at 4 °C in primary antibodies, washed with PBS and incubated in secondary antibodies overnight at 4 °C before cover slipping. Primary antibodies used were anti-TH (Abcam#ab76442; 1:500 dilution), anti-pS129 (Abcam#ab51253; 1:1000 dilution), anti-ubiquitin (Santa Cruz Biotechnology#sc-271289; 1:500 dilution), anti-IL-1β (Thermo Fisher Scientific#P420B; 1:250 dilution), anti-Lc3 (Cell Signaling Technology#83506; 1:500 dilution), anti-Lamp2a (Abcam#ab18528; 1:250 dilution) and anti-Iba1 (Synaptic Systems#234 308 Gp311H9; 1:500 dilution). The secondary antibodies used were Goat anti-Mouse IgG (H + L) Cross-Adsorbed, Alexa Fluor™ 488 (Thermo Fisher Scientific#A-11001); 1:1000 dilution, Goat anti-Rabbit IgG (H + L) Cross-Adsorbed, Alexa Fluor™ 488 (Thermo Fisher Scientific#A-11008); 1:1000 dilution, Donkey anti-Chicken IgY (H + L) Cross-Adsorbed, Alexa Fluor™ 568 (Thermo Fisher Scientific#A78950); 1:1000 dilution, Goat anti-Mouse IgG (H + L) Cross-Adsorbed, Alexa Fluor™ 405 (Thermo Fisher Scientific#A-31553); 1:1000 dilution, Goat anti-Rabbit IgG (H + L) Cross-Adsorbed, Alexa Fluor™ 647; 1:1000 dilution, and Goat anti-Guinea Pig IgG (H + L) Highly Cross-Adsorbed, Alexa Fluor™ 647, 1:1000 dilution]. The sections were imaged using a Zeiss Z.1 Light dual-sided illumination sheet fluorescence microscope, equipped with ZEN software. Quantification of pS129-syn, ubiquitin, LC3, and Lamp2a were performed blindly by identifying TH neurons in the red channel and measuring fluorescence in the green channel.

### Microglia analysis in mice

Images of mouse microglia were obtained using a 40x Z-stack confocal microscopy were processed and refined using ImageJ for further analysis. The “far red channel” images were used to define regions of interest (ROIs) for the corresponding microglia channel. Multilayer confocal images were compressed to generate projected microglia images. These images were then converted to binary and further processed with a median filter (radius = 2.0 pixels). To restore continuity in microglia structures fragmented by binarization, dilate and close operations were applied. ROIs from the far-red channel were applied to the microglia channel to establish brain region boundaries. Finally, individual microglia cells were analyzed using ImageJ’s “wand tracing tool,” measuring area, perimeter, and roundness within the ROIs.

### Biochemical fractionation and immunoblot

Extractions were performed using a modified procedure of Henderson et al. [20, 26]. Briefly, mouse brains (half) were homogenized in 9 volumes of high salt (HS) 1% Triton X buffer with protease and phosphatase inhibitors using Dounce homogenizers for 20 strokes. The samples were centrifuged at 100,000 x g for 30 min and supernatants were stored at -80. The pellets were homogenized in 9 volumes HS-Triton X buffer with 30% sucrose and centrifuged at 100,000 x g for 30 min at 4 °C. Supernatants were again stored at -80 °C. The pellets were homogenized in 9 volumes 1% Sarkosyl HS buffer, shaken for 30 min at room temperature (RT), and spun at 100,000 x g for 30 min. The resultant pellet was washed in 1.4 mL PBS without Ca^++^ or Mg^++^, resuspended in DPBS, and sonicated. An equal volume of supernatants from each extraction was combined and run on a 12% Bolt Bis Tris gel (Thermo Fisher Scientific#NP0321BOX) for 50 min at 150 volts, transferred to a nitrocellulose membrane, and blocked in 5% non-fat milk. Primary antibodies were diluted in 1% non-fat milk and are as follows: for pS129 (Abcam#ab51253; 1:2000 dilution), SQSTM1/p62 (Cell Signaling Technology#5114; 1:2500 dilution), LC3B (Cell Signaling Technology##3868; 1:2500 dilution), beclin1 (proteintech#11306-1-AP; 1:2500 dilution) and tubulin (Abcam#ab52866; 1:2500 dilution). Secondary donkey anti-rabbit horseradish peroxidase (HRP) (Santa Cruz Biotechnology#sc-2004; 1:2500 dilution) antibody diluted in 1% non-fat milk. Blots were developed in ECL Plus substrate for 5 min before imaging under Azure 300 - Chemiluminescent Imaging System and band intensity was quantified using ImageJ. The pSer129 blots were developed with SuperSignal Atto substrate.

### Cytokine assays

Enzyme-linked immunosorbent assays (ELISA) were used to quantify the following cytokines: IL-1α, IL-1β, TNF-α, IL-6, IL-10 and IFN-Ƴ levels in the soluble fractions from control and CPF-exposed mouse brains. The multiplex ELISAs were performed in triplicate by Quansys Biosciences (Q-Plex Mouse Cytokine Panel 1 HS, Logan, UT).

### ZF husbandry, strains and exposures

Transgenic lines and wild-type (AB) ZF were maintained at 28 °C, fed brine shrimp twice a day, and kept on regulated by 14/10 hours light/dark cycles. Fish eggs were obtained from natural mating, and embryos were collected and staged based on post-fertilization days. Transgenic ZF Tg(*vmat2*:GFP) [20, 27] expressing green fluorescent protein (GFP) driven by the vesicular monoamine transporter promoter (VMAT2) were used to identify aminergic neurons including DA neurons. Tg(*isl1*[ss]: Gal4-VP16, UAS: EGFP)zf154 [20, 28] transgenic line was used to visualize trigeminal and Rohon-Beard peripheral sensory neurons. To study microglial activation and autophagy, Tg(*mpeg1*:mCherry) [20, 29] and Tg(*elavl3*:eGFP:*map1lc3b*) [30] lines were utilized, respectively. All experiments were conducted following protocols approved by the Animal Research Committee at the University of California, Los Angeles. ZF embryos were manually dechorionated and were exposed to 250 nM CPF at 24 hpf at 28 °C for 4–6 days.

### ZF immunostaining and imaging

ZF larvae were anesthetized with 0.01% tricaine methane sulfonate (tricaine-S; Sigma-Aldrich#E10521), and fixed in 4% paraformaldehyde (PFA) overnight at 4 °C. The larvae were washed in 1X Dulbecco’s Phosphate Buffered Saline (DPBS, ThermoFisher Scientific #J67670.K2) and treated with 1 mL peroxide mix (3% H^2^O^2^ + 0.8% KOH in DPBS) for 8–10 min to bleach pigment. The larvae were washed with DPBS three times at RT immediately after the peroxide treatment and permeabilized for 5–7 min with 10 µg/mL Proteinase K (ThermoFisher Scientific #EO0491). Larvae were then washed in ddH2O and placed in a 10% blocking solution of 5% BSA + 5% goat serum for 1 h and incubated with primary antibody (anti-GFP; ThermoFisher Scientific #A11120, 1:500 dilution; anti-mCherry, Abcam #ab167453, 1:500 dilution; anti-TH, Sigma-Aldrich #MAB318, 1:250 dilution; or anti-Active Caspase-3, BD Pharmingen#559565, 1:500 dilution) in 1% BSA + 1% goat serum overnight at 4 °C. After five washes at 15-minute intervals, the larvae were incubated in secondary antibody overnight at 4 °C. After the secondary antibody washes, the samples were placed in 50% glycerol for 30 min and cleared in 100% glycerol. Larvae were dissected and mounted in 100% glycerol for imaging on the Leica SPE (Leica Microsystems Inc, Buffalo Grove, IL) confocal microscope. The *vmat2*:GFP positive neurons, TH neurons, microglia, and caspase-positive cells in the telencephalon and diencephalon regions were imaged in Z-stacks and analyzed. Peripheral sensory neurons were imaged and quantified by anesthetizing the transgenic *isl1*:GFP positive larvae and imaged at 10× magnification in the tail region and analyzed.

### γ1-syn knockout ZF

Exon 1 of the ZF *sncg1* gene encoding γ1-syn [20, 31] (also called *sncg1*) was targeted using a pair of custom transcription activator-like effector nucleases (TALENs; Supplemental Fig. 1) [32]. Possible target sequences were found using ZiFiT [33] and selected to: (i) encompass a unique restriction site that could be used to identify mutants and (ii) avoid off-target homology by BLAST search. DNA constructs encoding custom TALENs were generated by iterative assembly using the Joung Lab TAL plasmid set [20, 32]. The resulting TALEN plasmids were linearized and transcribed in vitro to generate mRNA (mMessage Machine T7, Invitrogen). Single cell ZF embryos were microinjected with 300 pg mRNA for each TALEN (total 600 pg) in 0.5–1.0 nL microinjection buffer. Surviving embryos were raised to sexual maturity and screened for the germline transmission of *sncg1* deletions by pairwise mating followed by PCR amplification and restriction digest of DNA from progeny embryos. Mating pairs with germline transmission were then outcrossed and their progeny were raised to adulthood. F1 founders were identified by fin clip PCR and the mutant allele was cloned and sequenced. We identified 3 different mutant alleles with deletions between 8-14 bp (Supplemental Fig. 1A). We selected a 13 bp deletion (allele designation Pt131) that is predicted to disrupt the *sncg1* open reading frame (Supplemental Fig. 1B) for further analysis. The line was expanded from a single F1 founder and outcrossed through four generations prior to analysis to remove any off-target mutations. Heterozygous in-crosses resulted in progeny genotypes at the predicted Mendelian frequency and the *sncg1*^*Pt131*^ mutation was stable over multiple generations. Homozygous *sncg1*^Pt131/ Pt131^ ZF were viable, fertile and showed no morphological or behavioral deficits during larval development. Rapid genotyping of the Pt131 allele exploited the deletion of a unique *Afe*I restriction site in exon 1 of sncg1 in the mutant (Supplemental Fig. 6C). Genomic DNA was extracted from adult fin clips or whole embryos and amplified using primers *sncg1*F 5’-CCTCTCTCTGTCATTGAAAC-3’ and *sncg1*R 5’-TAGGAAACACATGCACACAC-3’. The resulting 256 bp amplicon was then digested with *Afe*I (New England Biolabs), yielding bands of 164 bp and 92 bp for the WT allele, and a single band of 243 bp for the deletion allele (Supplemental Fig. 1C). Western blot analysis of adult brain homogenates showed the expected 14 kDa γ1-syn band in WT siblings but reduced expression in heterozygous *sncg1*^+/ Pt131^ ZF and complete loss of γ1-syn expression in homozygous *sncg1*^Pt131/ Pt131^ ZF (Supplemental Fig. 1D). These data confirm that Pt131 is a null allele. The homozygous mutant is correspondingly abbreviated to *sncg1*^−/−^ throughout the remainder of the paper.

### ZF behavior assay

ZF larvae were treated from 1 to 7 dpf with 250 nM CPF and washed with E3 egg water for 6 h prior to testing. Morphologically normal appearing ZF larvae (7 dpf) were transferred to 96-well plates with 16 larvae per condition, acclimated in the dark for 30 min, and their movements were monitored using a ZebraBox (ViewPoint ZebraLab, Civrieux, France). A 10-minute light/10-minute dark cycle was used for 3 cycles for a total of 1 h of recording. The distances moved by larvae during each increment of 10 min were averaged.

### LysoTracker labeling and microglial analysis in ZF

We used *LysoTracker* staining to image lysosomes in living ZF. Tg(*mpeg1*:mCherry) ZF (5 dpf) that have microglia labeled with mCherry, were incubated in the dark for 45 min using 10µM LysoTracker Green DND-26 (ThermoFisher Scientific#L7526) diluted in the treatment solution and then washed three times in E3 medium before mounting and imaging. Microglial structure was analyzed using ImageJ software as previously described [34, 35]. Briefly, Z-projected images were converted into a 16-bit grayscale file. The images were then made binary, and the skeletonization algorithm at FIJI was used for quantification.

### Measurement of autophagic flux

To measure autophagic flux in ZF neurons, we used transgenic [Tg(*elavl3*:eGFP:*map1lc3b*)] ZF larvae as previously described [30, 34]. Briefly, ZF larvae were treated with 250nM CPF between 24 and 120 hpf. The larvae were anesthetized with 0.01% tricaine and mounted with 1% low-melting agarose in a glass-bottom culture plate. eGFP-Lc3 punctate were counted with confocal imaging using a 40x oil immersion objective (numerical aperture = 1.15) and an excitation laser line of 488 nm. Z-stacks comprised 13 sections, and each section of punctate eGFP-Lc3 positive tectum regions was acquired with a slice thickness of 1.5 μm and 1024 × 1024 pixel resolution. For the flux assay, ZF larvae were incubated with 2µM BafA1 (Cayman Chemical, 11038) for 55 min and then immediately washed three times with E3 water prior to live imaging. For restoring autophagic flux, larvae were treated with 7.5µM calpeptin (Sigma-Aldrich#C8999) from 48 hpf to 7 dpf with solution refreshed daily.

### Western blots

For analysis of ZF proteins, approximately 40 brains were dissected and homogenized with Radio-Immunoprecipitation Assay (RIPA) lysis buffer with protease inhibitor. After homogenization on ice, samples were centrifuged and protein concentrations in the supernatant were determined using the Bicinchoninic acid (BCA) assay. A final volume of 25 µl was used to load and run 20–30 µg of protein on a 12% SDS-PAGE gel with 1-mercaptoethanol and 1x loading dye. Using XCell-II Blot Module (ThermoFisher Scientific#EI9051), proteins were transferred to the nitrocellulose membrane. Afterward, the transferred membrane was blocked in 5% non-fat milk for 2 h at RT and probed with anti-*sncg*1 (generated as described previously [34, 36]), anti-Sqstm1/p62 (Cell Signaling Technology#5114; 1:1000 dilution), anti-tubulin (Sigma Aldrich#T9026; 1:2500 dilution) primary antibodies at 4 °C overnight. Then the membranes were washed with Tris-buffered saline (50 mM Tris base, 150 mM NaCl, pH 7.5) with Tween-20 (Bio-Rad Laboratories, 1610781) (TBST) followed by donkey-anti-rabbit HRP (Santa Cruz Biotechnology#sc-2313; 1:2500 dilution), goat-anti-rabbit (Vector Laboratories Burlingame#BA-1000; 1:2500 dilution), and goat-anti-mouse HRP (ThermoFisher Scientific#62-6520; 1:2500 dilution) secondary antibodies. Bands were visualized by exposing the blots using chemiluminescent substrate ECL Plus (ThermoFisher Scientific#32132) and then quantified using ImageJ’s gel analysis feature.

### RNA extraction, cDNA preparation, and rtPCR analysis

RNA was extracted from ZF larvae at 5 days post fertilization using TRIzol reagent (Invitrogen, 15596026) according to the manufacturer’s instructions and converted into cDNA using iScript™ cDNA Synthesis Kit (Bio-Rad Laboratories#1708890). Gene-specific primer sets (*β-actin*F 5’-TTCCTTCCTGGGTATGGAATC-3’, *β-actin*R 5’-GCACTGTGTTGGCATACAG G-3’; *sncg1*F 5’-ATGGTGGTATGGAAGGAGGA-3’, *sncg1R* 5’-GGGCTCAGGGAAAGTCT TTT-3’; p62F 5’-GTCATATGGGTCCATCTCCAAT-3’, p62*R* 5’-AGGTGGGGCACAAGTCA TAA-3’) were used to conduct the real-time (rt) PCR reaction using SsoAdvanced Universal SYBR Green Supermix (Bio-Rad Laboratories#1725270) and cDNA. The 2^−ΔΔcT^ method was used to represent fold change values from cycle number data.

### Genetic Inhibition of microglial development and autophagy in ZF

To determine if microglia were contributing to aminergic neuron loss after CPF exposure, we used PU.1 targeted morpholino oligonucleotides (MOs) to inhibit microglial development. We targeted LC3 expression with MOs to determine if reduced autophagic flux can lead to aminergic neuron loss. Both MOs targeted the translational start codon (ATG) and were as follows: LC3, 5′-AGATCTGCCTAATTACTATCGTTTT-3′; PU.1 5′-GATATACTGATACTCCATTGGTGGT-3′; and scramble MO as a control 5′-CCTCTTACCTCAGTTACAATTTATA-3′ (Gene Tools, LLC). ZF embryos from 1- to 4-cell stages were injected into the yolk with MO stocks (1 mM) diluted 1:1 with 2% phenol red dye as previously described [34].

### Statistical analysis

As a part of the PEG analysis, we used univariate, unconditional logistic regression to estimate odds ratios (ORs) and 95% confidence intervals (CIs) for PD and CPF exposure separately by time window and location. As controls, we examined temporal trends in pesticide use based on age, gender, race/ethnicity, study wave, and index year (year of diagnosis or interview). Statistical analyses for mouse and ZF studies utilized the Student T-test and two-way ANOVA with the Bonferroni correction where appropriate and are listed in figure legneds. Error bars were presented as mean ± standard error of mean (S.E.M). The statistical significance level for all studies was set at *p* < 0.05.

## Results

### CPF exposure and its association with PD

To assess the impact of CPF exposure on PD risk, we utilized the Parkinson’s Environment and Genes (PEG) study and performed an updated analysis of a previous report that included more subjects and controlled for additional exposures (*n* = 829 PD patients; *n* = 824 controls) [34, 37]. PD patients were, on average, slightly older than controls, a higher proportion were men, had European ancestry, and were never smokers (Fig. 1A; Table 1). We estimated ambient exposure to CPF due to living or working near agricultural facilities applying CPF over a 30-plus year period. We observed positive associations between CPF and PD with exposure estimated at residential and workplace addresses and over different exposure time windows (Fig. 1D; Table 2). The strongest association was with the longest duration of exposure at the workplace, with an OR of 2.74 (CI 1.55, 4.89). Importantly, CPF exposures that occurred 10–20 years prior to disease onset were more strongly associated with PD than the 10-year period before PD onset. These associations were similar when adjusted for occupational pesticide exposures and other common ambient pesticide exposures (paraquat, glyphosate and diazinon; Supplemental Table 1).

**Table 2:** Chlorpyrifos risk estimated with logistic regression as ORs (95% CI) according to type of exposure assessments

Exposure Assessment and Time Window	PD patients (*n*=824)	Controls (*n*=821)	OR (95% CI)	*p*-value	PD patients (*n*=800)	Controls (*n*=782)	OR (95% CI)	*p*-value
	*n* (%) or Mean (SD)				*n *(%) or Mean (SD)			
Application Near:	Residence				Workplace			
**Any application, yes**								
1974-index year	499 (60.6)	452 (55.1)	1.35 (1.09, 1.68)	5.35E-03	415 (51.9)	347 (44.4)	1.39 (1.12, 1.73)	2.93E-03
20y - 10y prior to index year	394 (48.1)	336 (41.1)	1.47 (1.19, 1.82)	3.97E-04	321 (42.1)	248 (34.9)	1.39 (1.10, 1.74)	5.10E-03
10y prior to index year	382 (46.6)	337 (41.2)	1.26 (1.02, 1.55)	0.03	243 (36.3)	204 (34.4)	1.15 (0.89, 1.48)	0.28
**Application > Median in exposed controls, yes**								
1974-index year	267 (32.4)	226 (27.5)	1.35 (1.08, 1.70)	0.009474	224 (28.0)	173 (22.2)	1.39 (109, 1.79)	8.76E-03
20y - 10y prior to index year	218 (26.6)	168 (20.5)	1.58 (1.23, 2.03)	3.29E-04	164 (21.5)	124 (17.4)	1.34 (1.01, 1.78)	0.04
10y prior to index year	202 (24.6)	168 (20.5)	1.28 (1.00, 1.65)	0.05	140 (20.9)	102 (17.2)	1.28 (0.94, 1.75)	0.11
**Duration of exposure**, per one (equivalent to living near Chlorpyrifos application within buffer every year in window)								
1974-index year	0.17 (0.21)	0.14 (0.19)	2.68 (1.58, 4.55)	2.63E-04	0.14 (0.20)	0.11 (0.18)	2.74 (1.55, 4.89)	5.94E-04
20y - 10y prior to index year	0.22 (0.31)	0.18 (0.29)	1.99 (1.40, 2.85)	1.54E-04	0.19 (0.29)	0.16 (0.28)	1.61 (1.10, 2.38)	0.02
10y prior to index year	0.25 (0.34)	0.20 (0.32)	1.50 (1.09, 2.07)	0.01	0.21 (0.34)	0.18 (0.32)	1.33 (0.92, 1.91)	0.13
**Average exposure**, per 10 pounds Chlorpyrifos active ingredient applied per acre per year within buffer*								
1974-index year	3.0 (5.9)	2.2 (4.7)	1.39 (1.13, 1.71)	1.74E-03	2.6 (6.0)	1.9 (4.7)	1.35 (1.10, 1.67)	4.99E-03
20y - 10y prior to index year	4.1 (9.9)	3.1 (8.1)	1.19 (1.05, 1.34)	5.77E-03	3.7 (9.8)	3.0 (8.3)	1.10 (0.98, 1.25)	0.12
10y prior to index year	5.0 (12.2)	3.6 (9.2)	1.12 (1.01, 1.24)	0.03	4.8 (12.2)	3.4 (9.3)	1.15 (1.02, 1.29)	0.02
**Average exposure, log-transformed**, per one**								
1974-index year	0.7 (1.0)	0.6 (0.9)	1.22 (1.09, 1.36)	5.71E-04	0.6 (1.0)	0.5 (0.9)	1.22 (1.08, 1.37)	1.15E-03
20y - 10y prior to index year	0.7 (1.1)	0.6 (1.0)	1.20 (1.09, 1.32)	3.13E-04	0.7 (1.1)	0.5 (1.0)	1.13 (1.02, 1.26)	0.02
10y prior to index year	0.8 (1.2)	0.6 (1.1)	1.12 (1.02, 1.23)	0.02	0.7 (1.2)	0.6 (0.6)	1.12 (1.01, 1.24)	0.04

Models control for age, race/ethnicity, sex, index year, and study wave

*After outlier removal, the pounds of Chlorpyrifos applied per acre in any given year ranged from 0 to 62.0

**Average exposure (year average Chlorpyrifos applied per acre exposure assessment) was offset by 1 and log transformed. Values ranged from 0-4.1

### Effects of inhaled CPF on motor behavior in mice

**Fig. 2:**
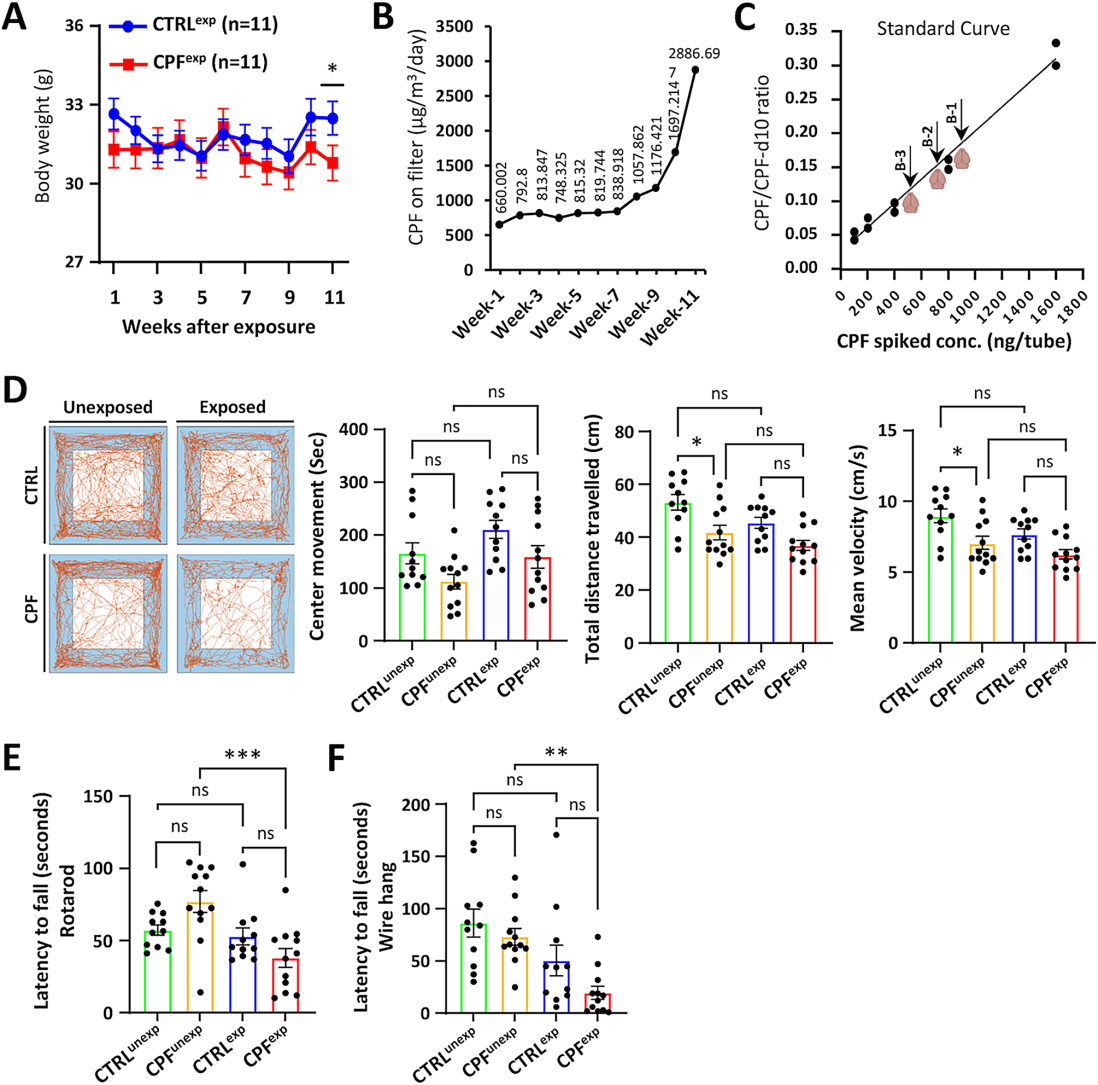
CPF exposure parameters and behavior assays on C57BL/6J mice. (**A**) Mice’s body weight was recorded before and during CPF exposure. (**B**) CPF concentrations in the exposure chamber over time. (**C**) CPF standard curve and mouse brain concentrations (arrows). (**D**) Open field performance. From left to right: Representative trajectories of individual control (CTRL) and CPF mice, values of total distance traveled, mean velocity, and center time movement. (**E**) Accelerating Rotarod performance and Wire hang test (**F**) latency to fall of control and CPF-exposed mice. Statistical significances represented with asterisks: ** = *p* < 0.01, and ns = not significant. P-values were calculated using two-way ANOVA with the Bonferroni correction (see Supplemental Fig. 2 for full statistical analysis)

Most human pesticide exposure is through inhalation, which escapes the 1st pass circulation to the liver with oral ingestion and therefore reduces its metabolism. To model human exposures, mice were exposed to aerosolized CPF or ethanol vehicle in closed chambers five days a week for 11 weeks. We found in preliminary experiments that female mice were much more resistant to CPF than males (data not shown), therefore, only male mice were tested in the current study. The concentrations of CPF used were determined empirically (the highest dose that was well tolerated), and it was increased over time as the mice adapted to the exposures. CPF-exposed mice maintained their body weight as did the ethanol vehicle controls until the last week of the experiment (Fig. 2A). The concentration of CPF in the chamber reached 650 µg/m^3^/day initially and peaked at 2900 µg/m^3^/day by week 11 (Fig. 2B). Concentration of CPF in the brains of exposed mice was measured by liquid chromatography-tandem mass spectrometry and reached 1.44 to 2.42 ng/mg tissue (Fig. 2C).

Mouse behavior was analyzed before the initiation of CPF exposures and 3 days following the last day of exposure using rotarod, wire hanging, and open field testing. The 3-day washout was used to ensure that the behavior was not altered by CPF or vehicle and likely reflected underlying pathology. The baseline measurements for the open field tests for the CPF group were different than the controls but both the exposed and control mice performed worse after 11 weeks (Supplemental Fig. 2). CPF-exposed mice deteriorated more than controls in both rotarod, and wire hang tests, but not in the open field test (Fig. 2D-F).

### CPF exposure leads to the loss of DA neurons

**Fig. 3:**
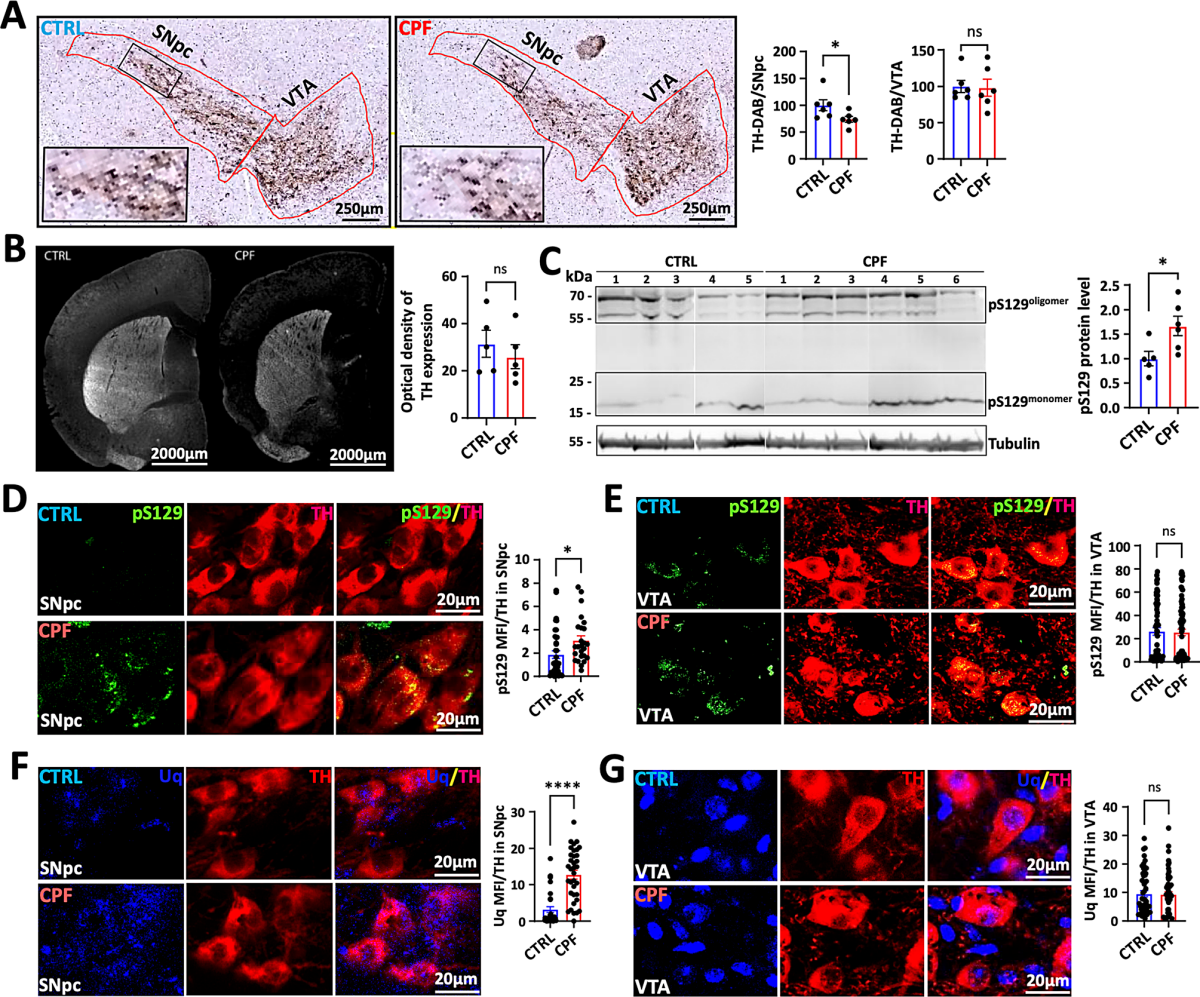
Effects of CPF inhalation on mouse brain pathology. (**A**) Immunofluorescence (IF) staining and histogram of the number of TH-positive cells in the SNpc and ventral tegmental area (VTA) of the mouse brain. Scale = 250 μm. CTRL: *n* = 5; CPF: *n* = 5. (**B**) Representative IF images of TH-stained striatum. Scale = 2000 μm. *p* = 0.06, Student’s t-test. (**C**) α-Syn at pS129 in insoluble fractions was detected by Western blot. (**D**, **E**) Representative SNpc and VTA images of pS129 α-syn positive aggregates in CPF-exposed mice. Scale = 20 μm. (**F**, **G**) Representative SNpc and VTA images with the histogram of ubiquitin staining. Scale = 20 μm. Statistical significances represented with asterisks: * = *p* < 0.05, **** = *p* < 0.0001, and ns = not significant. P-values were calculated using the two-tailed Student’s t-test. CTRL: *n* = 5; CPF: *n* = 5

A core pathological feature of PD is the relative selective loss of DA neurons, which results in impaired motor behavior. We determined the total number of DA neurons in control and CPF-exposed mice using stereological methods. CPF inhalation resulted in a 26% loss of tyrosine hydroxylase (TH) positive dopaminergic neurons in the SN compared to control mice. This DA neuron loss in the SN was selective, as CPF exposure did not affect the ventral tegmental area (VTA) DA neurons in comparison to control animals (Fig. 3A). Staining for TH in the striatum was reduced in the CPF-exposed brains relative to controls, consistent with DA neuron loss in the SNpc, but the difference did not reach statistical significance (*p* = 0.06, Fig. 3B). Phosphorylated α-syn at serine 129 (pS129) is a biomarker for pathological forms of α-syn and is elevated in PD brains. Western blot analysis confirmed that pS129 was significantly elevated 1.66-fold in the insoluble fractions of the CPF brains compared to controls (Fig. 3C, Supplemental Fig. 3) and a trend for increased pS129 protein level in the Sarkosyl soluble fractions (Supplemental Fig. 3). We found elevated levels of pS129 staining and ubiquitin in DA neurons of the SNpc compared to controls, whereas staining was similar in VTA neurons (Fig. 3D-G).

**Fig. 4:**
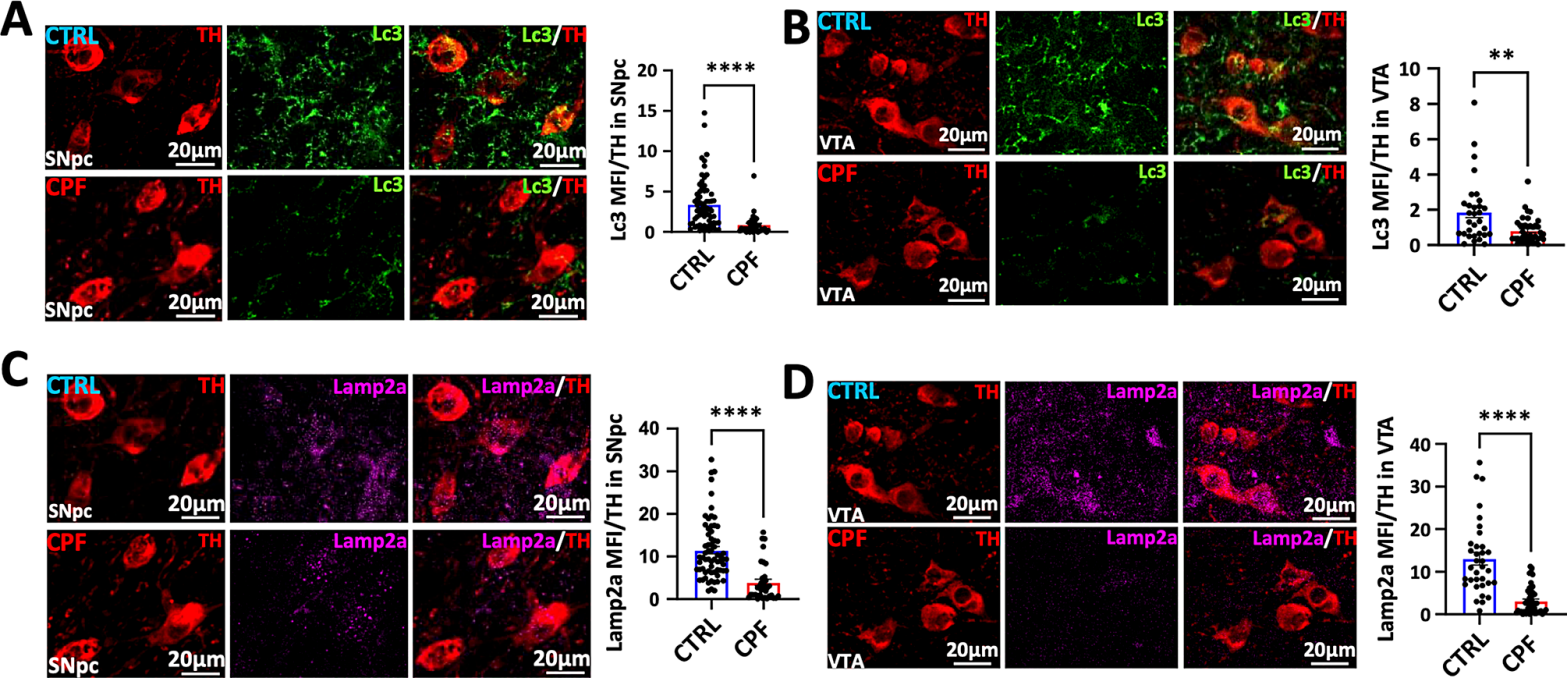
LC3 and Lamp2A immunohistochemistry in the SNpc and VTA of CPF-exposed mice. (**A**-**B**) Representative IF imaging of Lc3 (green) and TH (red) in the SNpc and VTA. Histogram bars indicated the mean fluorescence intensity (MFI) of Lc3 (green) in TH (red) positive DA neurons of C57BL/6J mice brain exposed to CPF. Scale = 20 μm. CTRL: *n* = 5; CPF: *n* = 5. (**C**, **D**) Similarly, IF images of SNpc and VTA sections stained with lysosome marker Lamp2a (magenta) and TH (red) in the SNpc and VTA. Histograms showed the lysosomal MFI in the brains of CPF-exposed and CTRL mice. Scale = 20 μm. CTRL: *n* = 5; CPF: *n* = 5. Statistical significances are represented by standard error of mean (SEM) with asterisks: **p < 0.01, and ****p < 0.0001. P-values were calculated using the two-tailed Student’s t-test

The increase in phosphorylated α-syn and ubiquitin in DA neurons in CPF-treated mice suggested that the accumulation might be due to dysfunction in protein degradation. Indeed, LC3-II and lamp 2a levels were reduced in TH-positive neurons in both the SNpc and VTA suggesting a defect in autophagy (Fig. 4). This defect does not appear to be widespread since Western blot analysis of LC3-II, p62 and Beclin1 in the soluble fraction from the CPF-treated whole brain homogenates did not show any differences from control fractions (Supplemental Fig. 4).

### Microglia take on an activated morphology after CPF exposure

**Fig. 5:**
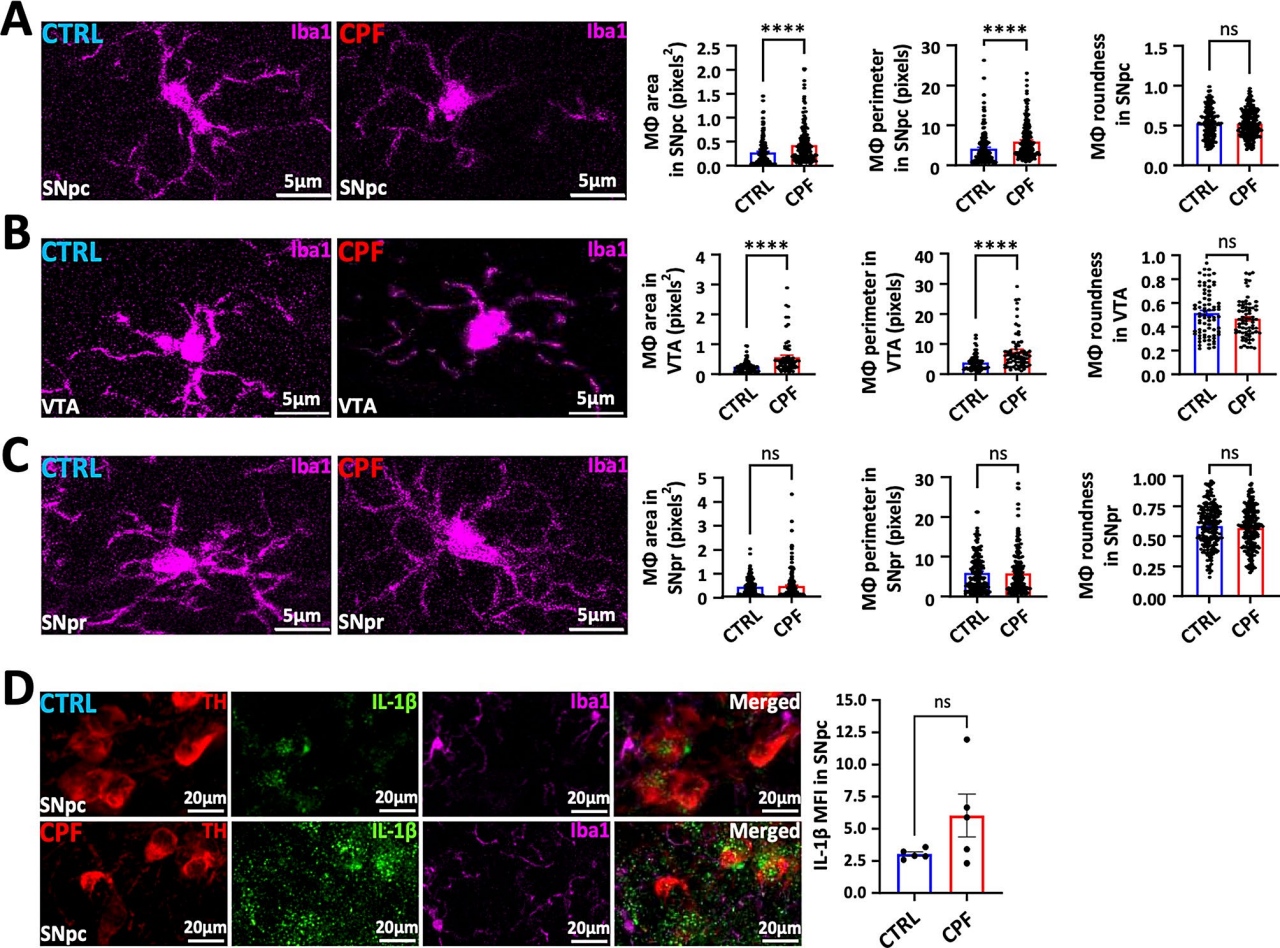
CPF-exposure causes microglia activation. (**A**-**C**) Representative confocal images of Iba1(magenta) staining used for microglia analyses at the SNpc, VTA, and SNpr. Images were converted to binary with randomly selected microglia. The histogram represented the microglial size (area and perimeter) and roundness. Scale bar = 5 μm. P-values were calculated using the two-tailed Student’s t-test. Statistical significance is represented by standard error of mean (SEM) with asterisks: ****p < 0.0001, and ns = not significant. CTRL: *n* = 5; CPF: *n* = 5. (**D**) Representative IF images of the proinflammatory cytokine IL-1β (green), Iba1(magenta) and TH (red) in CPF-exposed mice compared to the CTRL mice. The histogram showed the MFI of IL-1β in the SNpc. *P* = 0.1. Scale = 20 μm. CTRL: *n* = 5; CPF: *n* = 5. P-values were calculated using the two-tailed Student’s t-test

CNS inflammation is a common feature in PD and may contribute to its pathogenesis [34, 38]. Microglia change their shape and become more rounded in response to various stimuli such as environmental toxicants and infections [34, 38–40]. To investigate whether microglia take on an activated morphology after CPF exposure, we determined the mean size and perimeter of Iba1-stained cells using the method of Fernandez-Arjona et al. [41]. We found that CPF-exposed microglia were more rounded and had shorter projections in the SNpc and VTA but not in the SNpr (Fig. 5A-C). These morphological changes are consistent with activated microglia similar to those seen in PD brains. We tested the brain soluble fractions for the proinflammatory cytokines IFNү, IL-1α, IL-1ß, IL-6, and TNFα and the anti-inflammatory cytokine IL-10 by ELISA. All cytokines except IL-1α and IL-1ß were at the lower end of detectability and there were no statistical differences between the CPF-exposed brains and controls (Supplemental Figure 5). We did find a non-significant increase in IL-1ß in the SNpc region but not in the soluble fractions of whole brain homogenates (Fig. 5).

### CPF exposure induces selective neurotoxicity to DA neurons in ZF

We have found that exposure to CPF is associated with an increased risk of developing PD and that mice exposed to CPF through inhalation develop many PD features, including motor dysfunction, loss of DA neurons, α-syn pathology and activated microglia. To determine the mechanism of CPF neurotoxicity, we utilized transgenic ZF that are transparent and contain a well-developed DA system in the larval stage. Dechorionated ZF, 24 h post fertilization (hpf), were exposed to 0 to 100µM CPF for 6 days to determine its overall toxicity and found that all fish died at 30µM or higher by day 7 (Supplemental Fig. 6A, B**)**. To determine whether CPF is toxic to aminergic neurons, including DA neurons, we have utilized the transgenic (Tg) *vmat2*:GFP line that expresses GFP under the control of vesicular monoamine transporter 2 (*vmat2*) promoter to monitor aminergic (DA, noradrenergic, and serotonergic) neuronal integrity as previously described [34, 36, 42]. We used the lowest concentration at which we observed decreased locomotor activity (250 nM) for all subsequent experiments. A stereotypical pattern of swimming response to light was observed in vehicle-treated embryos and CPF-treated fish at 5 dpf at similar speeds but the CPF-exposed ZF swam slower than controls in the dark at 7 dpf (Supplemental Fig. 6C-F). This pattern of ZF behavior is consistent with DA neuron loss [34, 36].

**Fig. 6:**
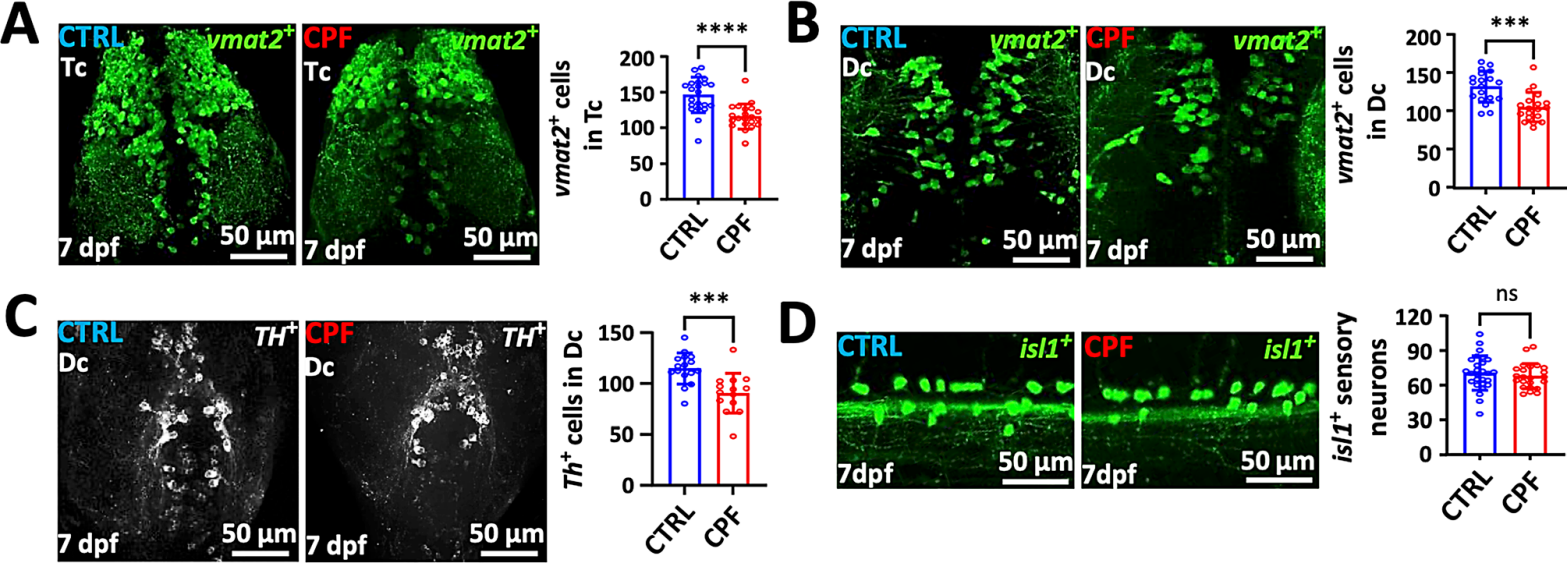
CPF induces selective loss of DA neurons in ZF embryos. (**A**-**C**) CPF exposure in Tg (*vmat2*:GFP) ZF larvae, and the counts of *vmat2*:GFP positive aminergic neurons and TH (red) positive DA neurons in the telencephalon (Tc), dorsal diencephalon (Dc) and ventral Dc, respectively. (**D**) CPF exposure in Tg (*isl1*:GFP) positive Rohon-Beard neurons (lateral view). Scale bar = 50 μm. Statistical analysis performed by a two-tailed Student t-test. Statistical significances represented with asterisks: *** = *p* < 0.001, **** = *p* < 0.0001, ns = not significant

The integrity of the aminergic system in ZF was determined at 5 and 7 dpf after CPF (250 nM) exposure. The number of aminergic neurons in the telencephalic (Tc), diencephalic (Dc), and TH positive neurons in the Dc clusters at 5 and 7 dpf ZF embryos were significantly reduced **(**Fig. 6A-C). Interestingly, increased apoptosis was observed in the telencephalic and diencephalic regions of 5 dpf embryos but not in the larvae at 7 dpf (Supplemental Fig. 7), suggesting that this is the time of active neuronal death. In order to test for selective DA neuron loss as seen in PD brains, we exposed 7 dpf Tg(*isl1*[ss]: Gal4-VP16,UAS: eGFP)^zf154^ larvae expressing GFP in Rohon-Beard neurons to 250 nM CPF and found no differences compared to controls at 7 dpf (Fig. 6D).

### Exposure to CPF activates microglia but does not contribute to neurotoxicity

**Fig. 7:**
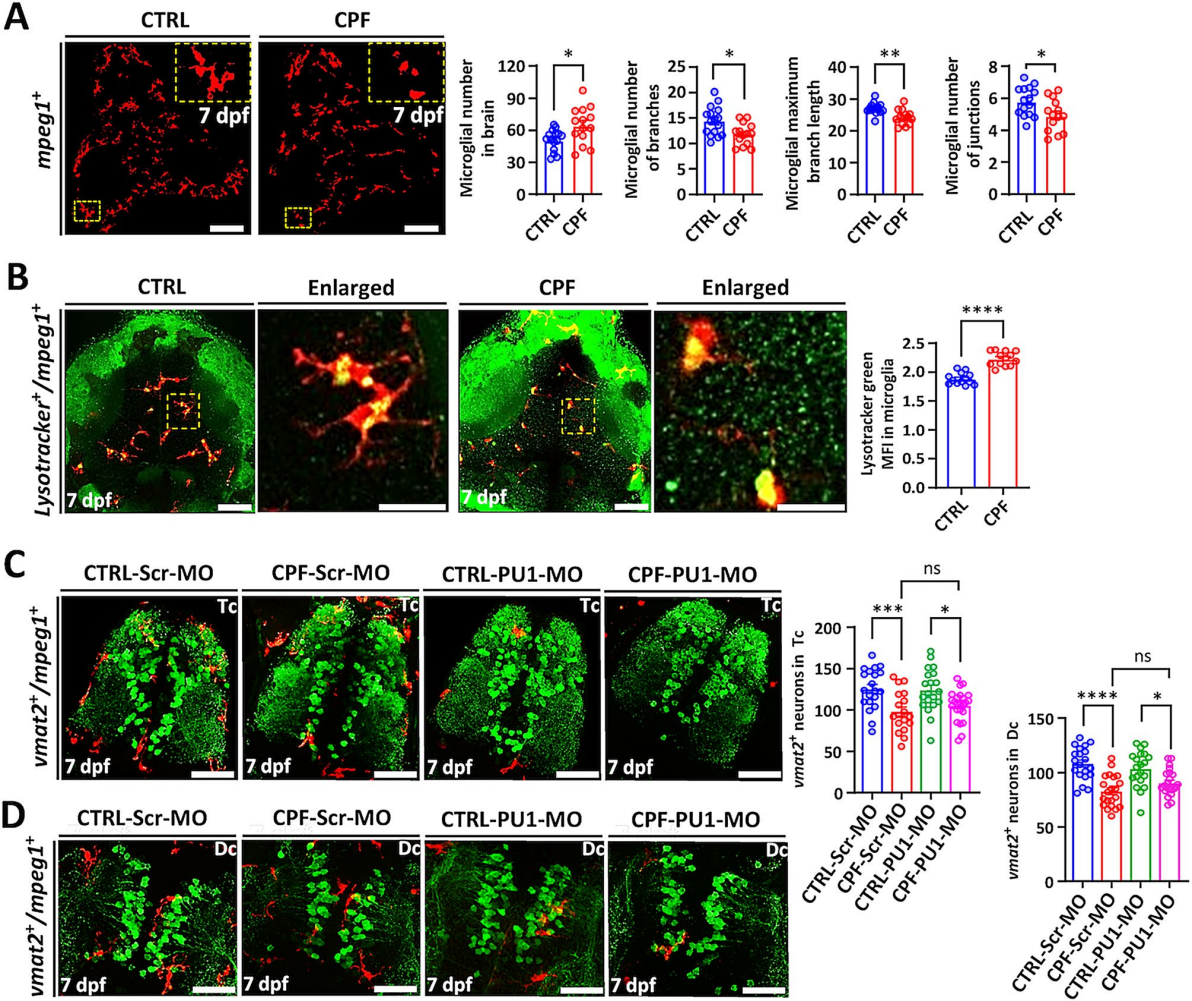
CPF neurotoxicity is not dependent on microglia in ZF. (**A**) Tg(*mpeg1:mCherry*) ZF embryos were treated with 250 nM CPF at 24 hpf for 6 days. Histograms indicated the number of microglia, microglial maximum branch length, number of branches and number of junctions in ZF larvae at 7 dpf. Scale bars: 75 μm. (dorsal view). (**B**) CPF-exposed Tg(*mpeg1:mCherry*) ZF larvae with lysotracker green staining. Histograms showed the lysotracker green MFI in the microglia (red). Scale bars: 25 μm, and 5 μm (Enlarged). Statistical analysis was performed using a two-tailed Student t-test. (**C**, **D**) Injection of PU1 morpholino oligonucleotide (MO) in Tg(*mpeg1:mCherry*) ZF embryos. The bar diagrams displayed comparisons between scramble (Scr) MO and PU1 MO with and without CPF. Scale bar = 50 μm. Statistical analysis was performed using two-way ANOVA with the Bonferroni correction. Statistical significances represented with asterisks: * = *p* < 0.05, ** = *p* < 0.01, *** = *p* < 0.001, **** = *p* < 0.0001, ns = not significant

Another pathological feature of PD is CNS inflammation, which we also found in the CPF-exposed mice. We utilized MPEG-mCherry transgenic ZF larvae that have red microglia and exposed them to CPF and found a higher number of microglia that were more rounded and had shorter processes at 7 dpf compared to controls **(**Fig. 7A**)**. Furthermore, the maximum branch length, the number of branches, and the number of junctions of microglia decreased. These structural changes in microglia are indicative of a more activated state, similar to that observed in mice. Activated microglia have more and/or larger lysosomes [43] so we used lysotracker labeling and live imaging to determine if the microglia were functionally activated. We found that the fluorescence intensity (MFI) of lysotracker in mCherry-labeled microglia was significantly increased following exposure to CPF consistent with microglia activation (Fig. 7B, Supplemental Fig. 8).

To evaluate whether microglia were involved in CPF-induced neurotoxicity, we inhibited microglial development using PU1 morpholinos (MO) before exposing the larvae to CPF. As shown in Fig. 7C and D, injection of PU1 MO resulted in marked reductions (87%-91%) of *mpeg1:mCherry*-positive microglia up to 7 dpf in both Tc and Dc regions of the brain. Neither scrambled control nor PU1 MO injections caused significant toxicity, nor did the injection significantly affect the number of surviving neurons in the embryos. Reduction of microglia had no significant effect on CPF neurotoxicity. Aminergic neurons were reduced by 22% in the telencephalon with no injection, 21% loss with control MO (scrambled) injection, and 14% loss with PU1 MO injection following CPF treatment (Fig. 7C). Similar results were found in the diencephalon (Fig. 7D).

### γ1-synuclein is necessary for CPF toxicity

**Fig. 8:**
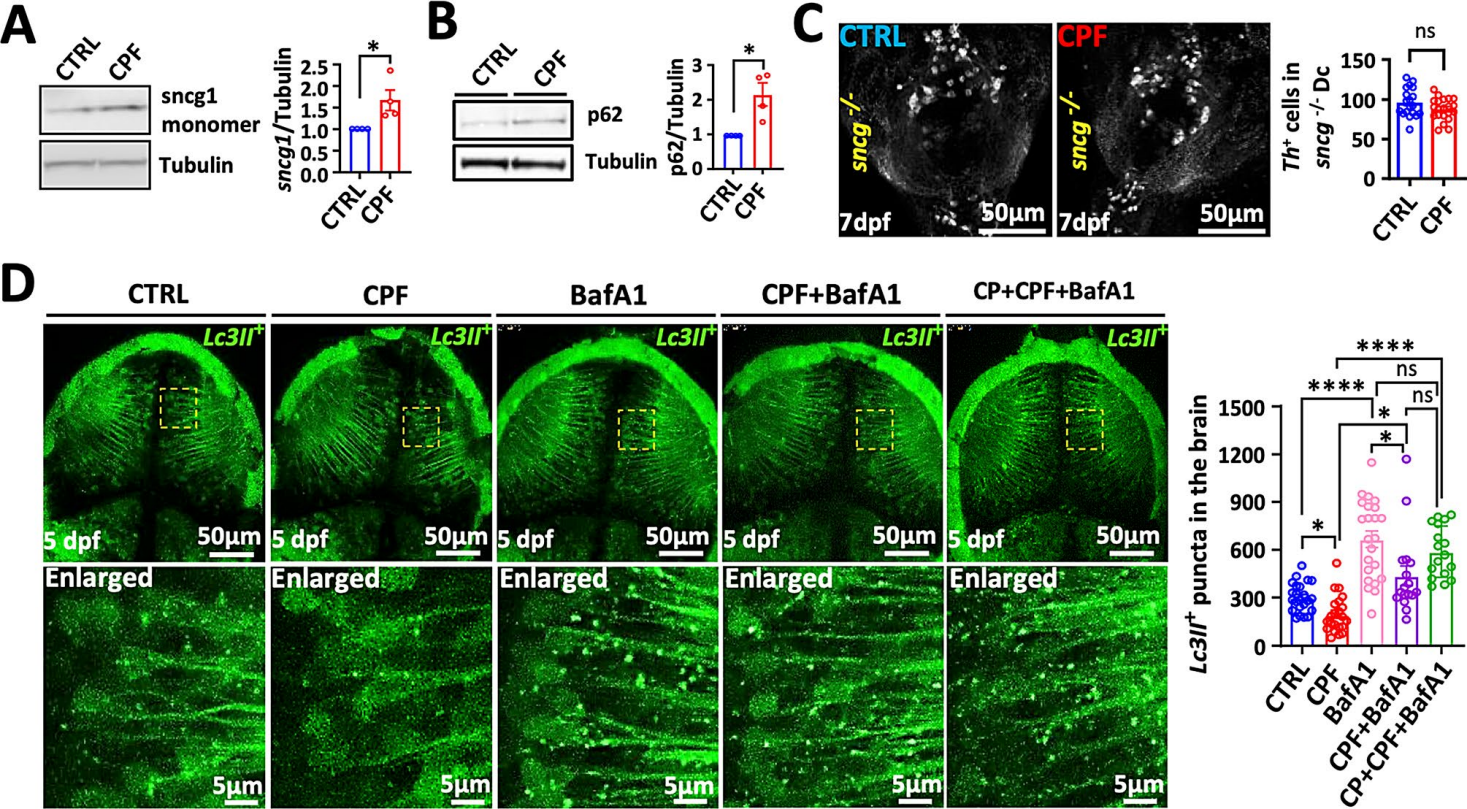
CPF reduces autophagic flux and neurotoxicity is dependent on ZF synuclein. (**A**-**B**) CPF treatment in ZF larvae and the detection of fish endogenous γ1-syn and p62 protein levels by western blot. (**C**) Representative Th (white) immunostaining in *sncγ1* knockout ZF larvae at 7 dpf. Scale bar = 50 μm. (**D**) The number of *Lc3II-*GFP positive puncta in the midbrain was counted in 5 dpf larvae with and without Bafilomycin A1 (BafA1). Statistical analysis was performed using two-way ANOVA with Bonferroni correction. Scale bar = 50 μm, and 5 μm (Enlarged). Statistical significances represented with asterisks: * = *p* < 0.05, **** = *p* < 0.0001, ns = not significant

Synucleins are neuronal proteins that comprise α-, β-, and γ-synucleins in mammals and α-syn aggregation and accumulation play a central role in PD pathophysiology [34, 44]. ZF do not contain an ortholog of human α-syn, although ZF γ1-synuclein (γ1-syn) has a similar function as α-syn [34, 45]. In order to determine if γ1-syn accumulates in CPF-exposed ZF, we performed Western blot analysis on dissected brain homogenates from 7 dpf larvae and found that γ1-syn protein levels were increased compared to controls (Fig. 8A), but mRNA levels were not, (data not shown). We then utilized γ1-syn knockout ZF to determine if the increase in γ1-syn contributed to DA neuron loss. CPF exposure did not result in DA neuron loss in γ1-syn knockout ZF suggesting that γ1-syn was required for CPF neurotoxicity in ZF (Fig. 8C).

### Reduced autophagic flux contributes to CPF neurotoxicity

**Fig. 9:**
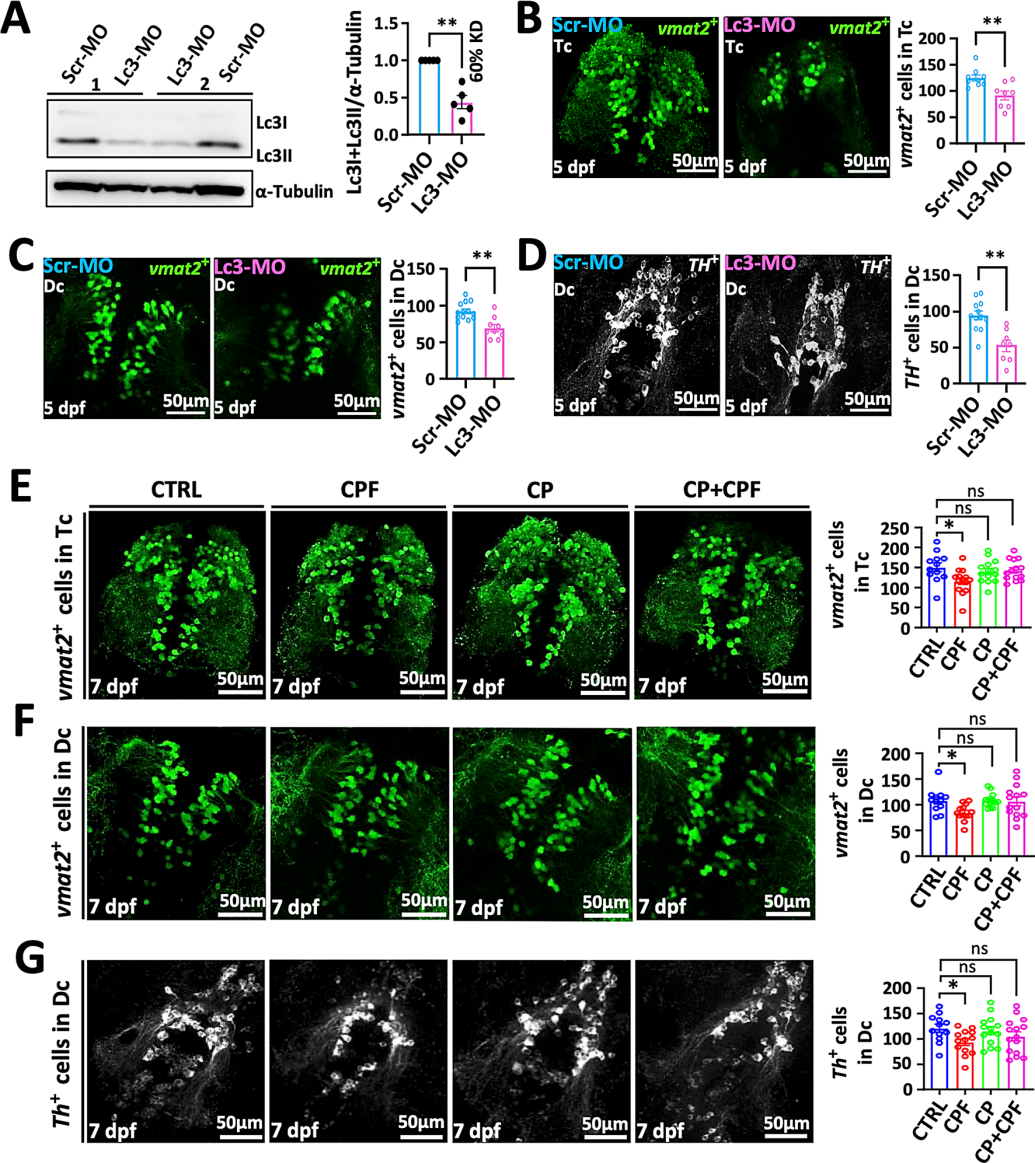
Reduced autophagic flux leads to DA neuron loss. (**A**) *Lc3* protein level at 5 dpf was reduced after MO knockdown ZF larvae. (**B**-**D**) Representative IF images of Tg (*vmat2*:GFP) larvae after Lc3 MO knockdown and the count of DA neurons in ZF’s Tc and Dc regions. Scale bar = 50 μm. Statistical analysis was performed using a two-tailed Student t-test, and statistical significances are represented with asterisks: ** = *p* < 0.01. (**E**-**G**). Representative IF imaging with and without calpeptin treatment revealed the number of *vmat2*-GFP-positive ammergic neurons and Th-positive dopaminergic neurons following CPF exposure. Scale bar = 50 μm. Statistical analysis showed the quantification of *vmat2*-GFP and Th-positive cells using ANOVA test with Bonferroni correction. Statistical significance represented with asterisks: * = *p* < 0.05, ns = not significant

CPF exposure led to an increase in γ1-syn protein levels but not its mRNA suggesting that the increase was due to decreased degradation. α-Syn is degraded by both the ubiquitin proteasome system (UPS) and autophagy but we focused on autophagy since aggregated α-syn is primarily degraded by this system [34, 46]. We utilized the Tg(*elavl3*:eGFP:*map1lc3b*) transgenic line to measure autophagic flux in neurons in vivo after CPF exposure and found a significant reduction in GFP-positive autophagosome (*Lc3-II*) puncta after treatment with CPF (Fig. 8D). We then measured the number of autophagosomes after 1 h of treatment with saturating concentrations of BafA1 and we found that CPF autophagic reduced flux (Fig. 8D). A reduction in autophagic activity by CPF exposure was further supported by the finding that the autophagy substrate p62 protein levels were increased while p62 expression was unchanged (Fig. 8B and Supplemental Fig. 9). Furthermore, knocking down LC3 using morpholinos reduced LC3 levels by approximately 60% and resulted in a significant decrease in DA neurons measured by both VMAT-GFP and TH IH (Fig. 9A-D). If disruption of autophagy contributed to DA injury, stimulating autophagy should be neuroprotective. We utilized calpeptin to induce autophagic flux (Fig. 9E-G and Supplemental Fig. 10), and we found that it rescued the neuron loss caused by CPF measured both by counting *vmat2*-GFP neurons and TH-positive neurons (Fig. 9E-G). Thus, CPF exposure led to reduced autophagic flux that contributes to DA neuron loss.

## Discussion

The goal of this study was to determine whether CPF exposure causes an increased risk of developing PD. In our extended community-based case-control study of PD in the central valley of California, we found that long-term exposure to CPF is associated with an up to 174% increased risk of developing PD. We also found both behavioral and pathological features of PD in CPF-exposed mice including motor dysfunction, DA neuron loss, pathological α-syn, and neuroinflammation. Furthermore, we found that the mechanism of CPF neurotoxicity includes decreased autophagic flux and the accumulation of pathological α-syn, both of which were required for CPF-induced DA neuron loss. Taken together, these findings strongly support a causal association between CPF exposure and PD.

There are several strengths to this study which include the use of the PEG cohort and geocoded exposure assessment. This exposure assessment is based on the Pesticide Use Report database in California, which provides detailed data on agricultural pesticide use since 1972, and lifelong residential and workplace address histories for all study participants. We estimated cumulative exposure for each study participant based on their home and work proximity to agriculturally applied CPF. Our approach has been well validated and is routinely used for air pollution research [18, 34]. A long lag time between exposure and the diagnosis is important since it is believed that the α-syn pathology begins decades prior to the core symptoms of PD become apparent. The association of CPF exposure with PD was stronger for exposures in the 10–20 years prior to the diagnosis and overall strongest with the longest duration window (Table 2). Importantly, exposure assessment did not rely on a subject’s recall which is often inaccurate and subject to recall bias. Another strength of the PEG studies is that all PD diagnoses were confirmed by a movement disorders specialist who examined each subject, often multiple times, over several years. One of the difficulties in researching ambient pesticide exposure in agricultural regions is the large number of pesticides applied. CPF application does not occur in isolation. We performed a series of co-exposure adjustments, however, and saw very similar results (Supplemental Table 1). In addition, our findings are consistent with our previous studies [34, 47, 48], and another study that similarly reported increases in risk after CPF exposure [34, 49].

We next set out to determine if the association of CPF with increased risk of developing PD is biologically plausible using two different animal models. Previous studies have reported loss of TH and dopamine neurons following oral and intraperitoneal CPF exposure but this is the 1st study that tested the primary means by which humans are exposed, inhalation [12–14, 50]. Inhalation of toxicants avoids the portal blood flow system to the liver that occurs with oral ingestion and, therefore, is not metabolized as rapidly. We exposed mice to inhaled CPF and found that it induced behavioral deficits, DA neuron loss, pathological α-syn, and inflammation, all characteristics of PD. Both the CPF-exposed and control mice’s’ behavioral tests worsened over time presumably due to aging or possibly the stress of the exposures, but the CPF group decreased more. The lesions in the DA pathway were only reduced by 26%, which may explain why we did not see alterations in the open field behavioral test. Mice were exposed for only 11 weeks, and longer exposures would likely result in more severe pathology.

It is very difficult to determine an exposure concentration that is relevant to human exposures. CPF plasma concentrations of 90 nM were measured in human volunteers [51] and environmental exposure to CPF in US adults and children were estimated to be 1.4–1.61 µg/kg/day [52] so the concentrations used in our studies are only a little higher and therefore relevant to human exposures.

The solubility of CPF in aqueous solution was a limitation on the concentration we could aerosolize. We therefore dissolved it in 75% ETOH which alone did not have overt effects to the mice. In preliminary dose finding studies, 3-month-old female mice showed no behavioral effects to CPF concentrations (1.7–1.9 mg/m^3^/day) which was over 100-fold lower than the LD50 for inhaled CPF previously reported [53]. When we started with 6-month-old males, half of them died in less than 24 h. at the same level of exposure. The concentration was therefore reduced to 1/3 of this concentration and gradually increased as tolerated until we noted the beginning of weight loss and ended the study. Increased vulnerability in older male mice to CPF exposure is consistent with the gender and age bias seen in humans [6, 53].

We also found increased phosphorylated α-syn in DA neurons in the CPF mice relative to controls, which was confirmed by Western blot. Although Sarkosyl insoluble phosphorylated α-syn was increased, Syn 506 antibody labeling did not significantly stain the CPF-exposed brains (data not shown). Syn 506 is a monoclonal antibody that recognizes some misfolded fibular α-syn [53, 54]. More time might be necessary for more complex α-syn aggregates to form and/or microglia may have been able to clear them.

An important component of establishing biological plausibility is determining the mechanisms by which a toxicant acts. We utilized ZF for this purpose since they are transparent, easily manipulated genetically, and contain a well-developed DA system even in the larval stage. We found that CPF exposure at very low concentrations (250 nM) caused selective toxicity to DA neurons. This toxicity was at least partially due to γ1-syn since its levels were increased and DA neurons were unaffected when CPF was tested in γ1-syn knockout fish. The elevated levels were the result of decreased degradation since expression was unchanged. We found that CPF exposure resulted in reduced autophagic flux that contributed to CPF-induced DA neuron loss given that stimulating it was protective against CPF neurotoxicity. This was further confirmed by reducing LC3 expression using MOs which also resulted in loss of DA neurons. It is difficult to study autophagic flux in mice, but we did find that phosphorylated α-syn was increased and the autophagy markers LC3-II and Lamp2a were decreased in the SNpc consistent with the ZF data. Furthermore, there was a trend for higher p62 and lower LC3-II in the supernatant from the CPF-exposed mouse brains consistent with the reduced autophagic flux measured in the ZF. Since many of the alterations we found with CPF exposure were concentrated in the SNpc, it is not surprising that the changes were diluted when testing a whole brain homogenate.

Previous studies have reported that CPF causes DA neuron loss in rodents [12–14, 50]. , but Singh et al. performed a detailed study of the mechanism of CPF toxicity in cell lines and rats [13]. They described motor deficits and loss of TH protein after oral administration of CPF that was caused apoptotic neuron loss via activation of a mitochondrial-mediated oxidative stress mechanism. They did not describe a clear reduction in autophagy, but they did report increases in LC3 and p62 with CPF exposure. There were several differences in our studies that can explain some of the different results including differences in the models, methods of exposure, and concentrations of CPF. Singh et al. used 10 µM CPF for their mechanistic studies in cell lines since this was the lowest concentration that induced apoptotic cell death while we used an intact vertebrate model (i.e. ZF) using 250 nM CPF for our mechanistic studies so we would not expect to cause mitochondrial toxicity. Singh et al. administered CPF to their rats orally which would lead to increased bioactivation by the liver than in our inhalation model. Despite these differences, there were some important similarities in our findings. We found that the autophagic cargo protein p62 was increased as did Singh et al. Our results differ in that LC3-II was decreased in both of our models while they found an increase. Lower levels of LC3-II would be expected if autophasomes were being formed at a reduced rate leading to elevated levels of p62 due to reduced degradation as we found. Higher levels of LC3 could represent an increase in autophasome formation in response to activation of the apoptotic pathway or a reduction in fusion to lysosomes such as occurs with the addition of bafilomycin. We did not find any changes in beclin in whole brain homogenates in contrast to Singh et al. which may reflect that disturbances in autophasome formation occurred downstream of beclin or that changes in a selected neuron population was diluted in the whole brain homogenate. Despite these differences, both studies consistently found that CPF exposure leads to DA neuron loss and alterations in autophagy.

Despite the finding that microglia took on an activated morphology in both mice and ZF, microglial activation did not appear to significantly contribute to DA neuron loss in ZF since reducing their development with morpholinos had little effect on CPF neurotoxicity. We attempted to characterize the cytokine profile in mice but unfortunately, we could not determine if the microglia were pro-inflammatory because our methods were not sensitive enough to accurately measure them in mouse brain homogenates except for IL1-α and IL1-β where there were no differences. We did find a trend for an increase in IL1-β in the SNpc which suggest a pro-inflammatory state, but additional studies would be necessary to fully characterize the microglia responce. Nevertheless, microglia may contribute to pathology in more chronic exposure models and in aging human brains.

There are some weaknesses to the ZF model. They are developing at the time of exposure, whereas PD is a disease of the aged. We do not think this invalidates our results since younger animals generally are more resistant to toxicants relative to older animals, suggesting that we may be underestimating CPF toxicity, not overestimating it. Since the primary purpose of using the ZF was to determine the mechanism of toxicity, we believe this weakness does not alter the validity of our findings.

## Conclusions

Exposure to CPF, a widely used pesticide, is associated with an increased risk of developing PD in humans. Mice exposed to CPF, in a similar manner to humans, developed motor deficits and pathological characteristics of PD. CPF appears to exert its neurotoxicity by reducing autophagic flux that results in increased α−syn. Taken together, CPF increases the risk of developing PD, and its use and exposure in humans should be restricted. Furthermore, stimulated autophagy might be an important therapeutic target in modifying disease progression.

## Supplementary Information

Below is the link to the electronic supplementary material.

Supplementary Material 1

## Data Availability

No datasets were generated or analysed during the current study.

## Abbreviations

α-syn: α-synuclein
CPF: Chlorpyrifos (O,O-diethyl O-3,5,6-trichloro-2-pyridyl phosphorothioate)
DA: Dopaminergic
Dpf: Days post fertilization
γ1-syn: γ1-synuclein
GFP: Green fluorescent protein
MO: Morpholino
PD: Parkinson’s disease
PEG: Parkinson’s Environment and Genes
pS129: Phosphorylated α-syn at serine 129
SNpc: Substantia nigra pars compacta
TH: Tyrosine hydroxylase
VTA: Ventral tegmental area
ZF: Zebrafish
