# Supplementary material for: The pesticide chlorpyrifos increases the risk of Parkinson’s disease
Source: Mol Neurodegener. 2025 Dec 11;21:3. doi: 10.1186/s13024-025-00915-z (PMC12801438; doi:10.1186/s13024-025-00915-z)
Supplement: Supplementary file 1 — Supplementary Material 1 [file 13024_2025_915_MOESM1_ESM.docx]

# **Supplemental tables and figures:**

**Supplemental Table-1.**

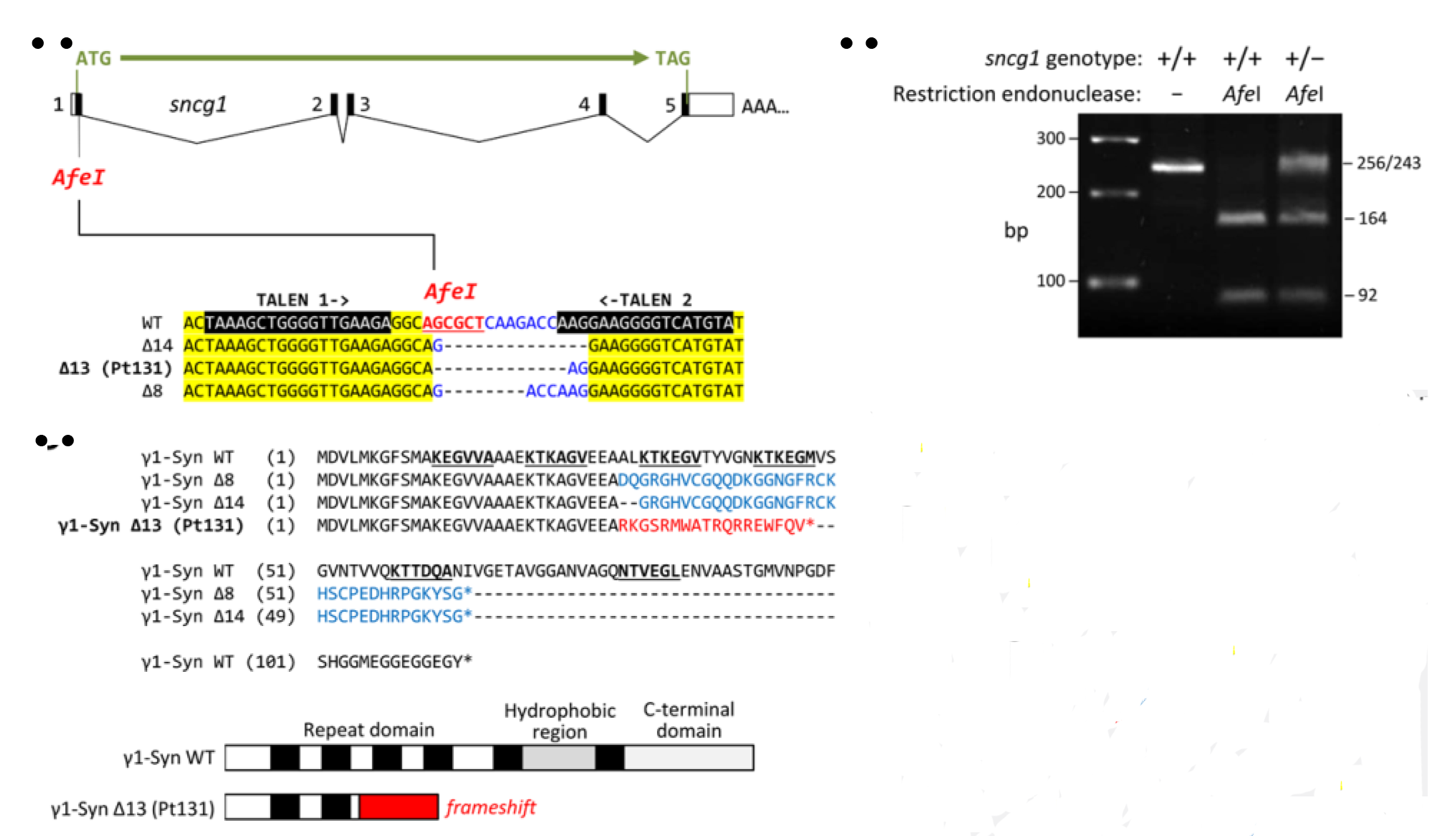


**Supplemental Figure 1. γ1-syn knockout ZF.** (A) γ1-syn knockout ZF was generated using TALENs to target an *AfeI* restriction site in exon 1 of the sncg1 gene. The upper part of the panel shows the genomic structure and open reading frame of the sncg1 gene and mRNA. The sequence alignment below shows the positions and sequences of the two custom TALENs, and the three small deletion alleles found in F1 founders. (B) The alignment shows the amino acid sequence of γ1-syn with the predicted effects of each of the three deletion alleles shown in panel A. The γ1-syn KTKEGV imperfect repeat sequences are underlined, and the frame shift translations of the mutants are shown in blue (frame +1) or red (frame +2). The cartoon below summarizes how the Pt131 mutation truncates γ1-syn near the N-terminus. (C) Ethidium-stained 2.5% agarose gel showing PCR products from genomic DNA derived from WT (+/+) or heterozygous (+/−) Pt131 mutants, amplified using *sncg1*-specific primers, and then incubated with *Afe*I or no enzyme. The WT amplicon of 256bp is cleaved by AfeI into two bands of 164bp and 92bp. The Pt131 mutation removes the *Afe*I site, so there is an uncut band of 243bp in addition to the WT restriction fragments in the heterozygous sample. Please see Supplemental Figure 9 for Western blot of ZF brain. The Pt131 mutation abolishes expression of γ1-syn , confirming this is a null allele


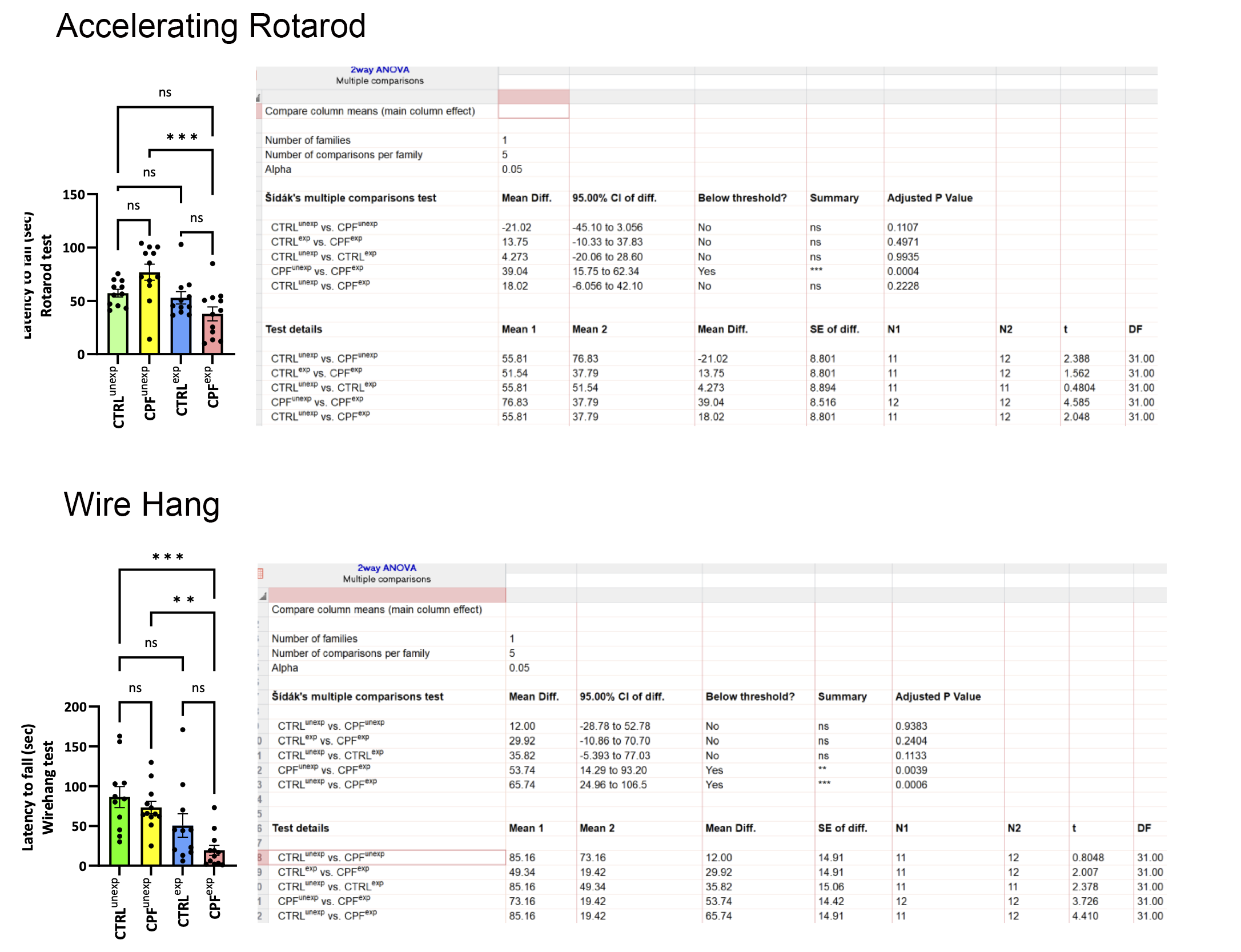


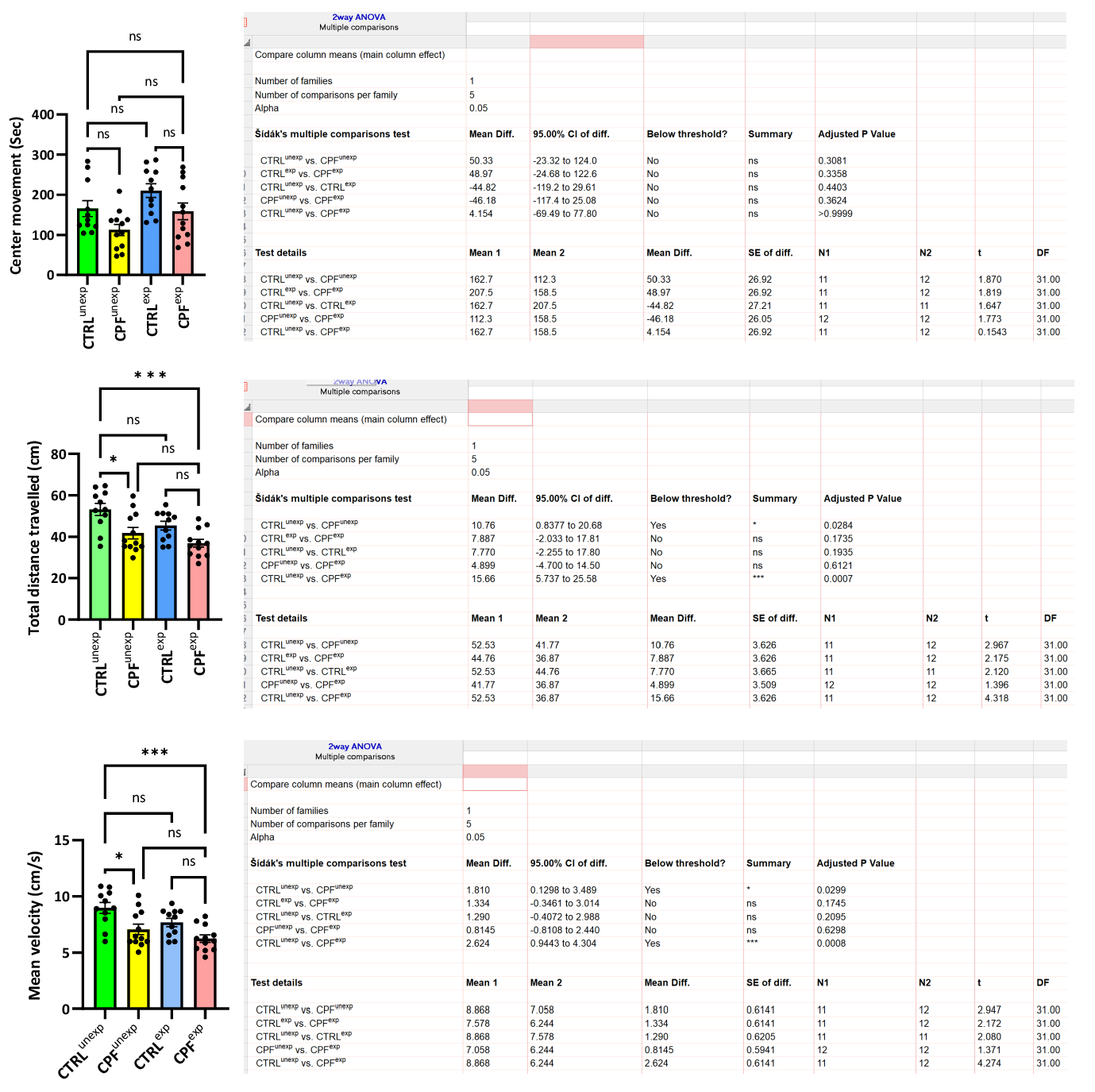


Open Field

**Supplemental Figure 2**. Full statistical analysis of mouse behavior using two-way ANOVA with the Bonferroni correction.


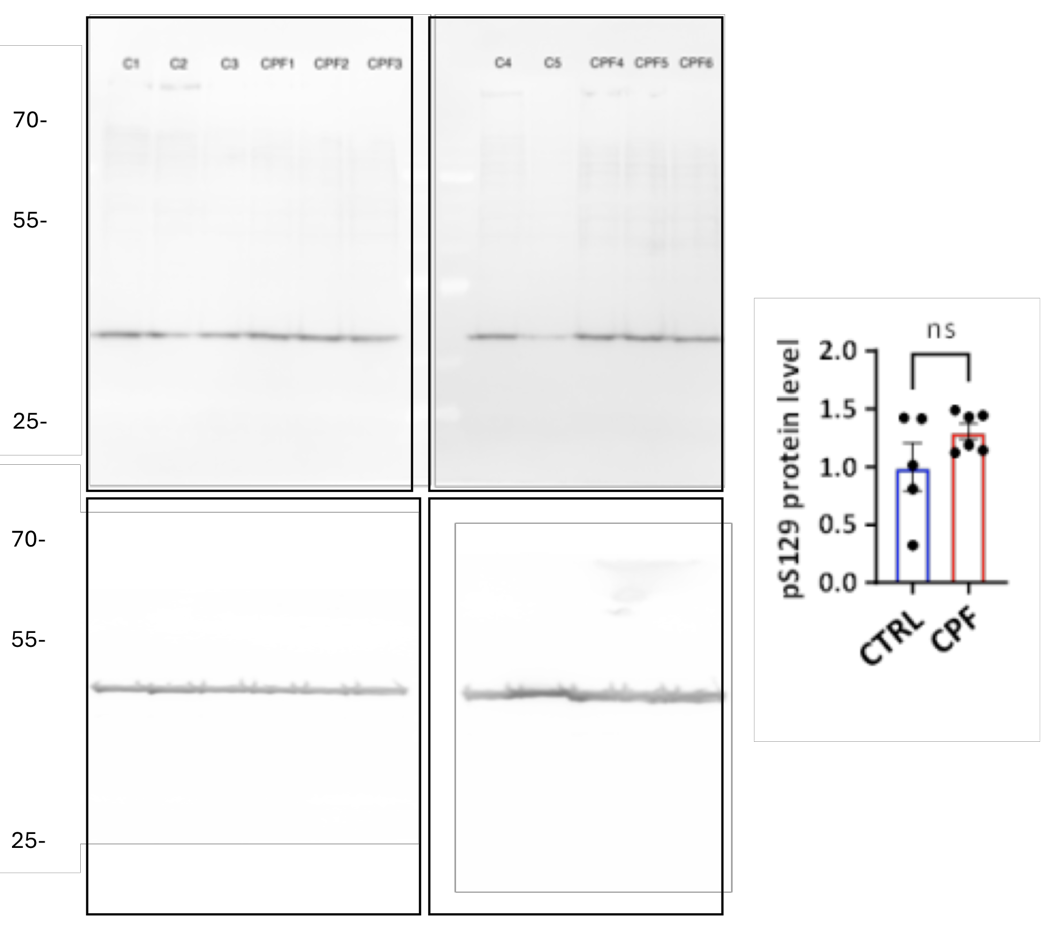


A


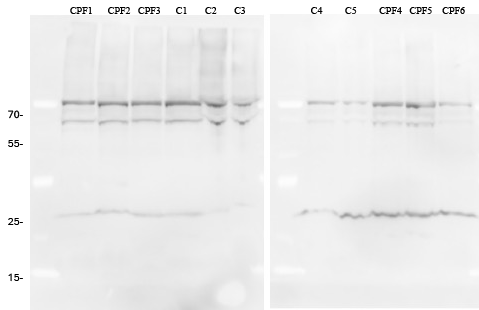


C

B

**Supplemental Figure 3**. Western blots of soluble α-Syn pS129 (A, arrow) and tubulin (B). Full Western blot of insoluble fraction from Figure 3 shown in panel C (arrows refer to oligomers, arrow head refers to monomer0. CPF=chlorpyrifos ,C = control; P = 0.06 for soluble fraction, Student’s t-test. Error bars = standard error of mean (SEM). ns = not significant.


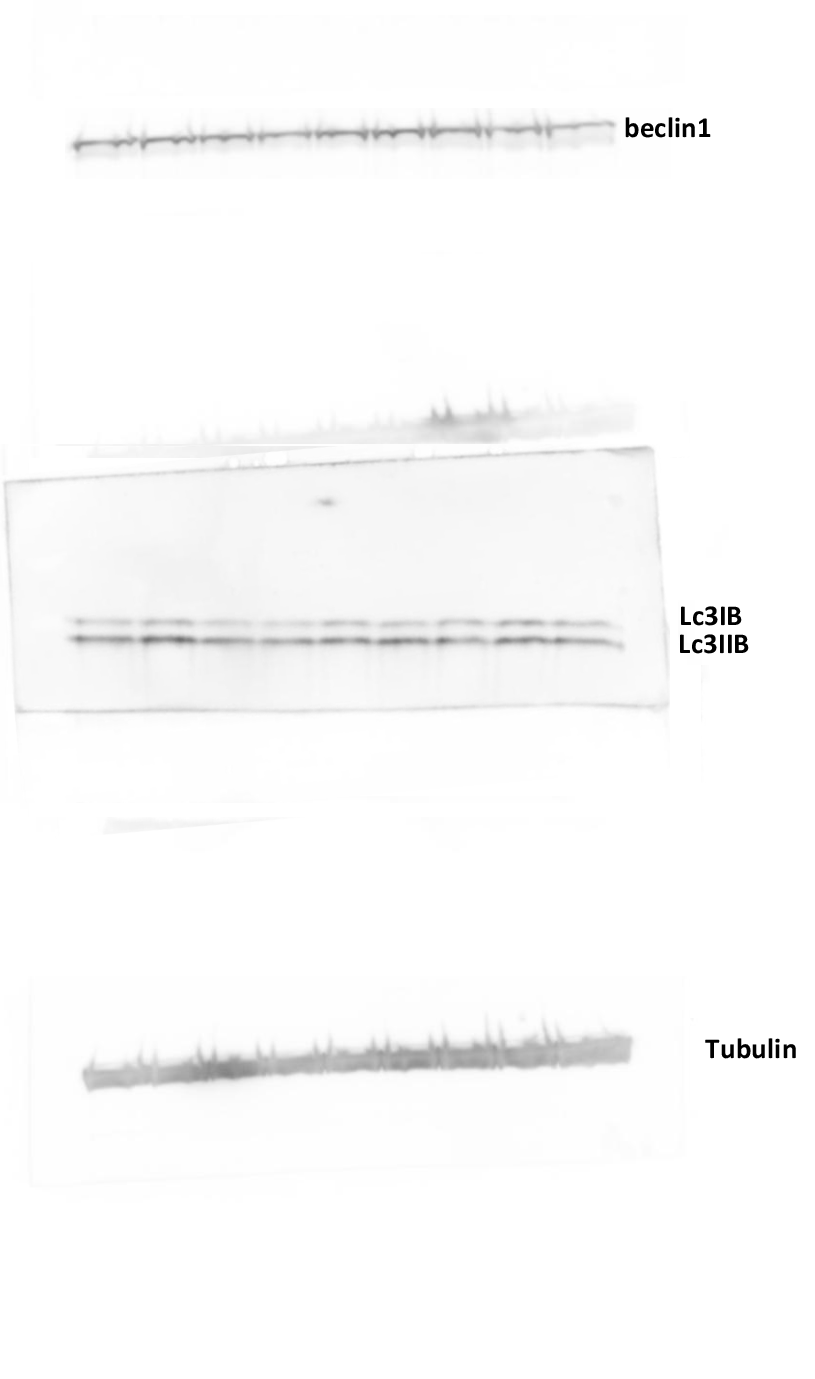

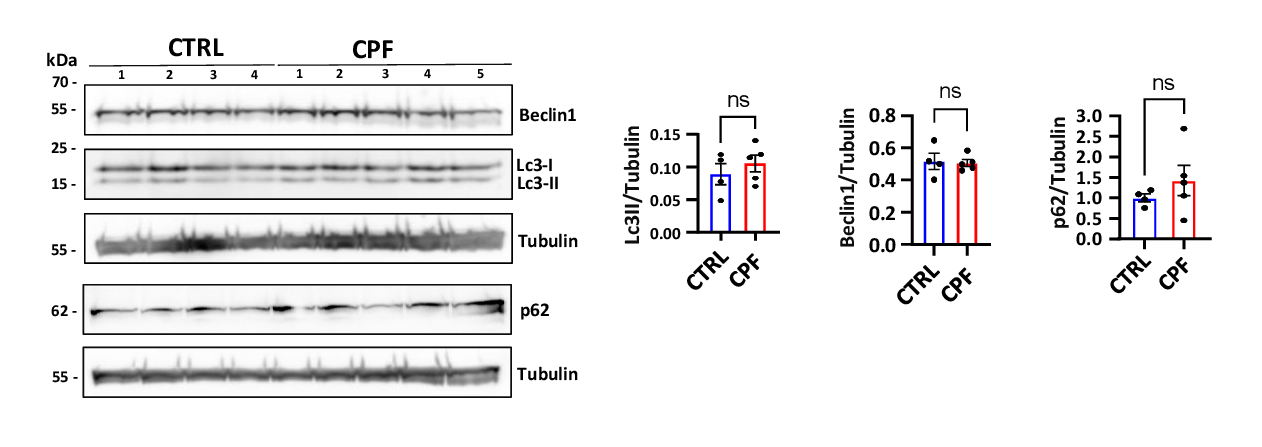


**Supplemental Figure 4.** Western blots of beclin, LC3 p62 from soluble fractions of mouse brain homogenates. p62 (arrow, top blot). CTRL = control; CPF=chlorpyrifos. Tubulin loading controls are shown. Full blots shown below. Student’s t-test. Error bars = standard error of mean (SEM). ns = not significant.

**Supplemental Figure 5.** ELISA results from soluble fractions of control (CTRL) and chlorpyrifos-exposed brains. A) IL-1β, B) TNFα, C) IL-1α, D) IL-6, E) IFN γ, F) IL-10 (only 3 samples had detectable levels). Student T test ns = not significant.


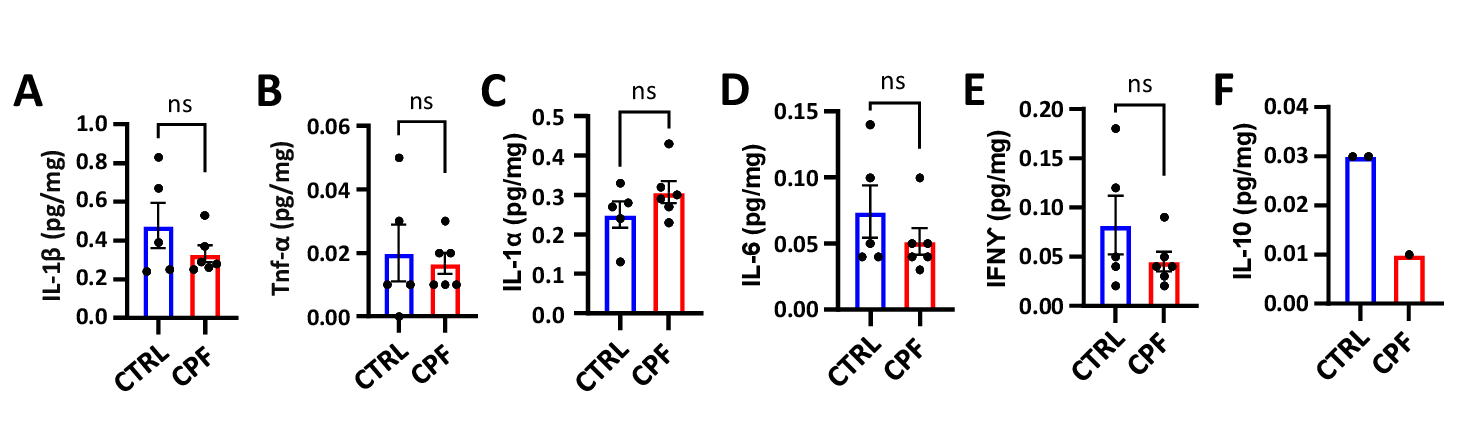


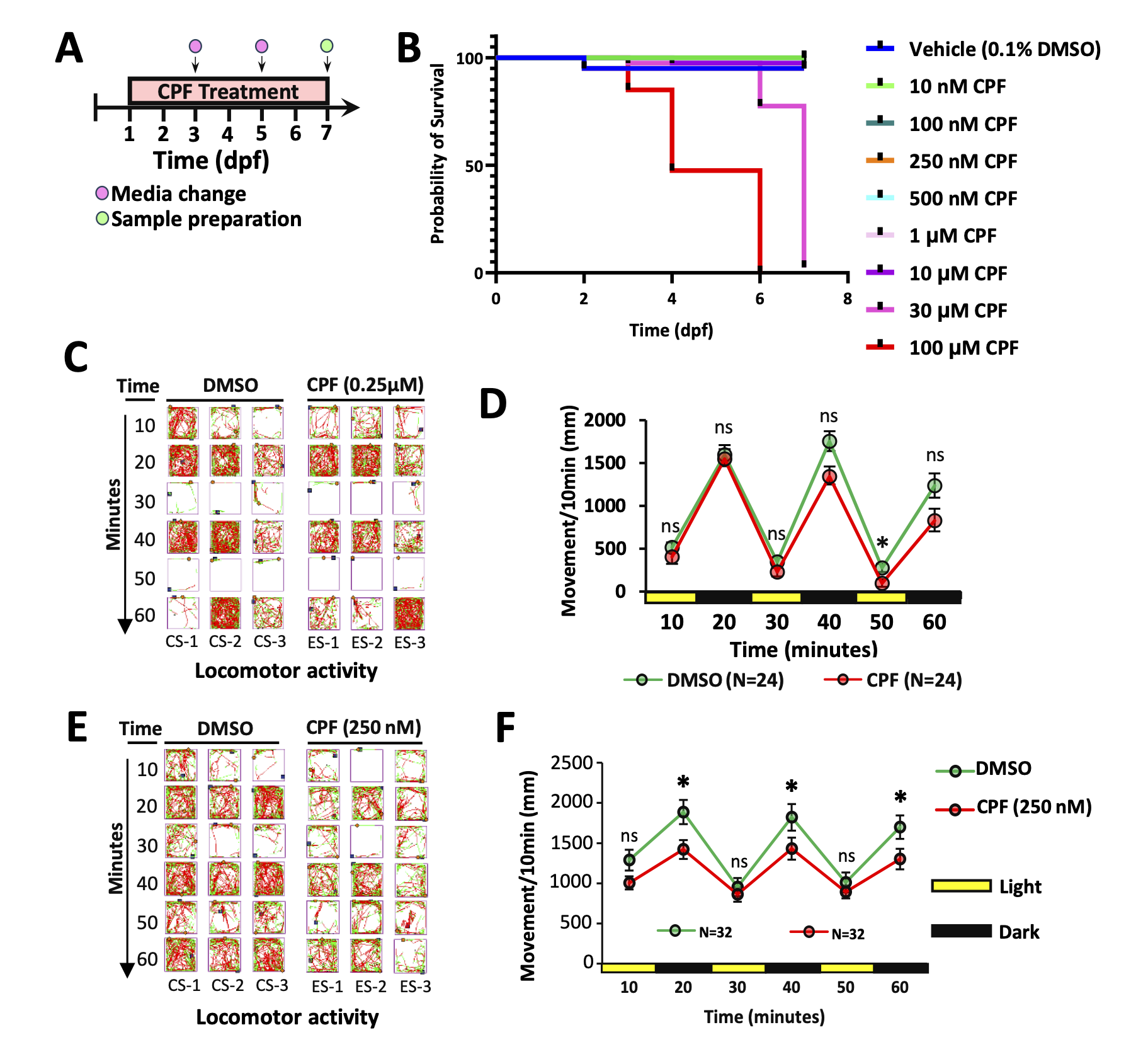


**Supplemental Figure 6. Pesticide treatment and behavior assay in ZF larvae.** (A) Tg(*vmat2*:GFP) embryos were exposed to CPF at 24 hpf until 7 dpf, replacing the water every other day (B) Embryos were treated with different doses of CPF (10 nM to 100 µM), and no lethality was observed until 30 µM, as indicated by the survival curve. Motor behavior (distance > 2mm) was tracked under an alternating light (yellow)/dark (black) cycle. (C and D) No significant differences were recorded in 5 dpf CPF-treated ZF larvae but 7 dpf larvae traveled significantly less during periods of darkness than vehicle-treated larvae (E,F). Statistical analysis was performed by two-tailed, Student’s t-test. * = p < 0.05, ns = not significant. All error bars are presented as the standard error of mean (SEM).


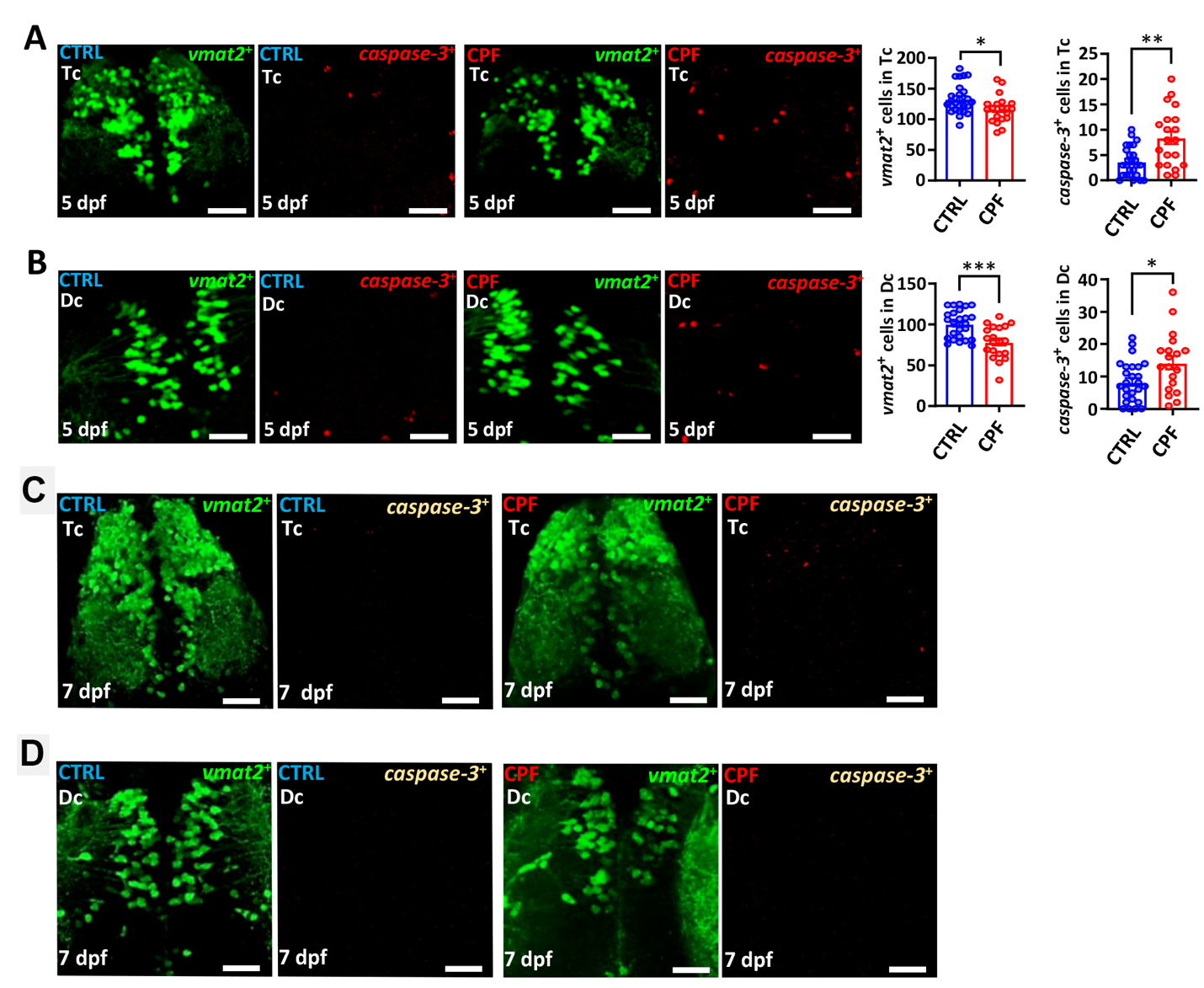


**Supplemental Figure 7. CPF induces apoptosis in ZF larvae.** (A, B) Representative images of *caspase-3*-stained ZF larvae showing that CPF induces apoptosis in the Tc and Dc at 5 dpf. The number of *caspase-3* positive cells in the midbrain was counted based on Z-Stack (20 layers; each of 2µm thickness) images. Statistical analysis quantified the numbers of *vmat2-*GFP positive telencephalic and diencephalic clusters of 5 dpf larvae**.** No caspase-3 positive cells were seen in 7 dpf exposed larvae (C and D). Scale bar = 50 μm. Statistical analysis performed using Student T-test. * = p < 0.05, ** = p < 0.01, *** = p < 0.001. All error bars are presented as the standard error of mean (SEM).


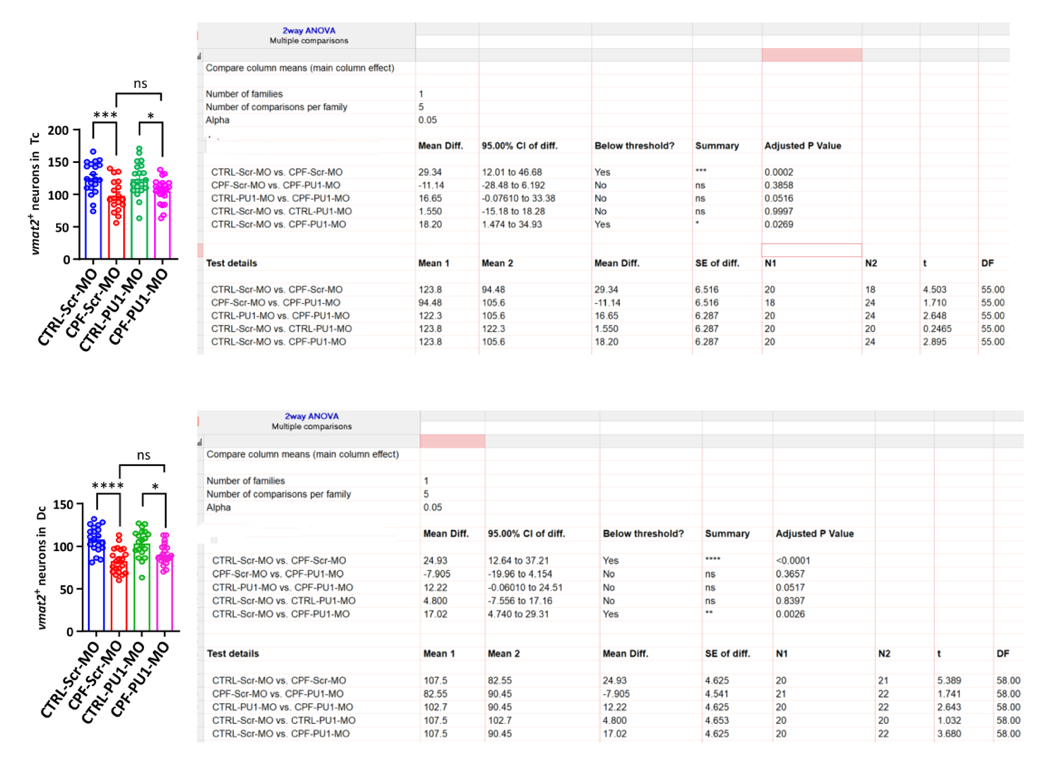


**Supplemental Figure 8.** Statistical analysis of MO knockdown of PU.1 using ANOVA with the Bonferroni correction.


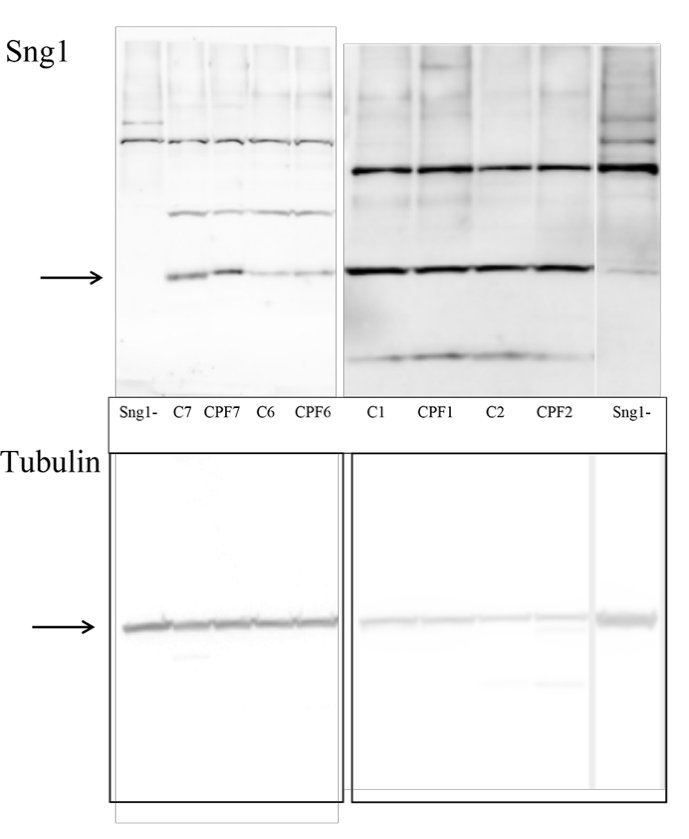

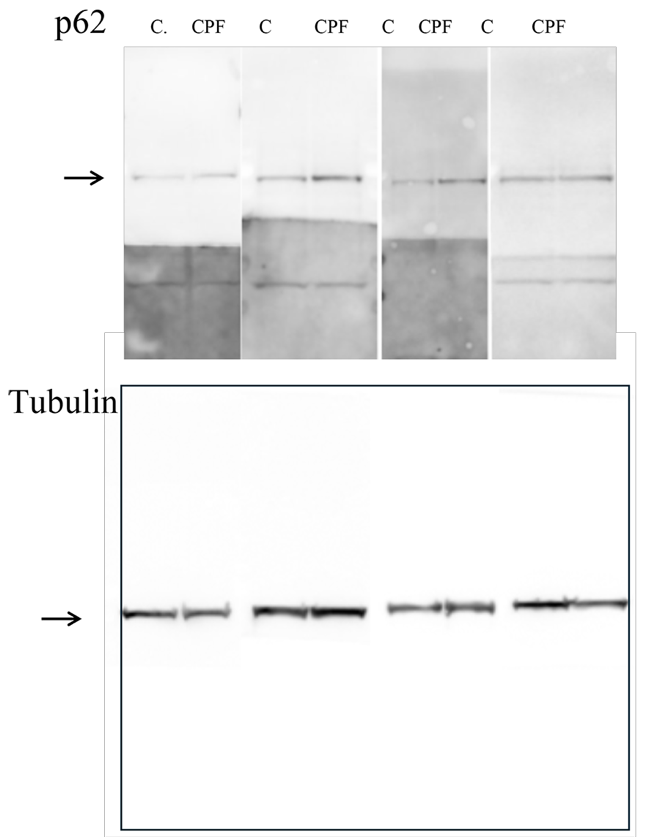


**Supplemental Figure 9.** Western blots of γ1-syn (arrow, left blot) and p62 (arrow, right blot) in ZF. C = control; CPF=chlorpyrifos; γ1-syn - = γ1-syn 1 knockout fish. The 14 KD protein is absent in the knockout ZF (arrow). The high MW band is non-specific. Tubulin loading controls are shown in lower panel.

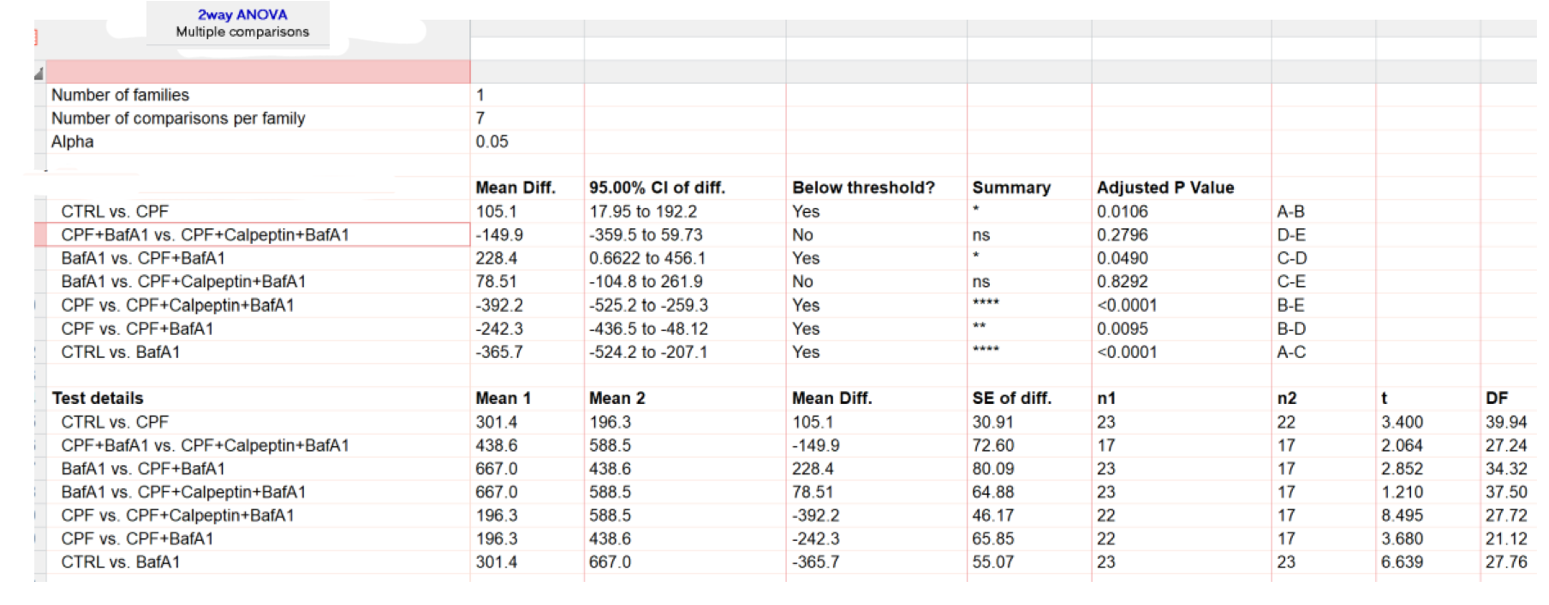


**Supplemental Figure 10. Autophagy induction with calpeptin increases autophagic flux in ZF larvae.** Representative images of *Lc3II-*GFP positive cells in the optic tectum regions of 5 dpf larvae. The number of *Lc3II-*GFP positive puncta in the midbrain was counted based on Z-Stack (10 layers out of 15 layers) images. Statistical analysis showed the quantification of the numbers of *Lc3II-*GFP positive puncta in the optic tectum of 5 dpf larvae performed by two-way ANOVA with Brown-Forsythe and Welch’s multiple comparisons tests. Scale bar = 50 μm, and 5 μm (Enlarged). CP: Calpeptin. * = p < 0.05, **** = p < 0.0001, ns = not significant. All error bars are represented as the standard error of mean (SEM).
